# Structural basis for conformational equilibrium of the catalytic spliceosome

**DOI:** 10.1016/j.molcel.2021.02.021

**Published:** 2021-04-01

**Authors:** Max E. Wilkinson, Sebastian M. Fica, Wojciech P. Galej, Kiyoshi Nagai

**Affiliations:** 1 MRC Laboratory of Molecular Biology, Francis Crick Avenue, Cambridge Biomedical Campus, Cambridge CB2 0QH, UK

**Keywords:** splicing, RNA, spliceosome, ATPase, cryo-EM, thermodynamic equilibrium

## Abstract

The ATPase Prp16 governs equilibrium between the branching (B^∗^/C) and exon ligation (C^∗^/P) conformations of the spliceosome. Here, we present the electron cryomicroscopy reconstruction of the *Saccharomyces cerevisiae* C-complex spliceosome at 2.8 Å resolution and identify a novel C-complex intermediate (C_i_) that elucidates the molecular basis for this equilibrium. The exon-ligation factors Prp18 and Slu7 bind to C_i_ before ATP hydrolysis by Prp16 can destabilize the branching conformation. Biochemical assays suggest that these pre-bound factors prime the C complex for conversion to C^∗^ by Prp16. A complete model of the Prp19 complex (NTC) reveals how the branching factors Yju2 and Isy1 are recruited by the NTC before branching. Prp16 remodels Yju2 binding after branching, allowing Yju2 to remain tethered to the NTC in the C^∗^ complex to promote exon ligation. Our results explain how Prp16 action modulates the dynamic binding of step-specific factors to alternatively stabilize the C or C^∗^ conformation and establish equilibrium of the catalytic spliceosome.

## Highlights

• Cryo-EM reveals new C_i_ spliceosome intermediate between branching and exon ligation
• Binding of branching and exon-ligation factors to C_i_ governs spliceosome equilibrium
• Exon-ligation factors Slu7 and Prp18 bind C_i_ weakly before Prp16 action
• After Prp16 action, pre-bound Slu7 and Prp18 bind strongly to promote exon ligation

## Graphical abstract

**Figure:**
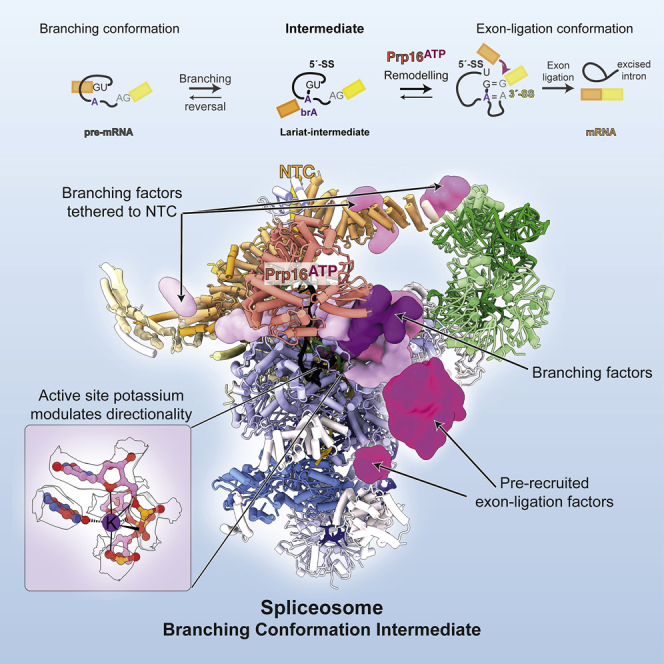

## Teaser

Genetic and biochemical studies suggested that the two steps of pre-mRNA splicing are in thermodynamic equilibrium through two major conformations of the spliceosome during catalysis. Here, the structure of a new spliceosome intermediate between these two conformations reveals how the binding of step-specific protein factors establishes equilibrium of the spliceosome.

## Introduction

The spliceosome produces mRNA by excising introns from pre-mRNAs in two sequential phosphoryl transfer reactions—branching and exon ligation. The spliceosome assembles *de novo* on each pre-mRNA through protein and RNA interactions that recognize conserved sequences at exon-intron junctions, called splice sites (Wilkinson et al., 2020). The U6 small nuclear RNA (snRNA) recognizes the 5′-splice site (5′-SS), while the U2 snRNA pairs with the intron around the branch adenosine (brA), forming the branch helix (Fica et al., 2017; Wilkinson et al., 2020). The 5′-exon is stabilized in the active site by pairing with loop I of the U5 snRNA (Newman and Norman, 1992; Sontheimer and Steitz, 1993). The U2 and U6 snRNAs fold into a triple helix conformation that constitutes the active site and allows U6 snRNAs to position two catalytic metals (Fica et al., 2013, 2014; Wilkinson et al., 2020). This active site is stabilized by the binding of the Prp19-associated complex (Galej et al., 2016; Wilkinson et al., 2020) (NTC). The NTC may also serve as a hub for the recruitment of some step-specific splicing factors (Chiu et al., 2009; Liu et al., 2007b).

**Figure 1:**
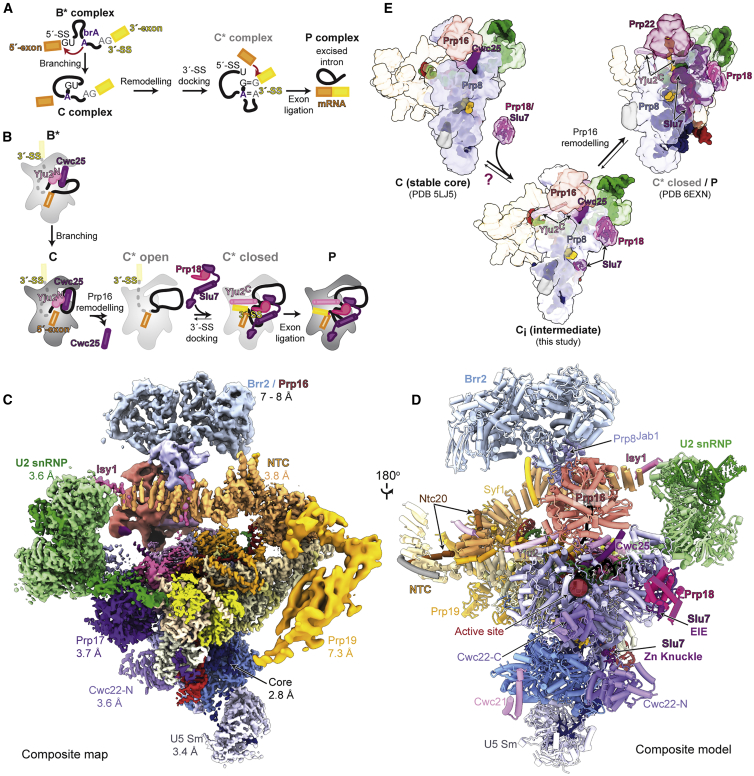
A novel C-complex spliceosome intermediate (A) Pre-mRNA splicing mechanism. (B) Canonical pathway for binding of exon ligation factors during the catalytic stage of splicing. (C) Composite cryo-EM map of the C-complex spliceosome. Average resolution (Fourier shell correlation [FSC] = 0.143) is indicated for each focused map. (D) Composite model of the C complex with pre-bound exon ligation factors. (E) The novel C-complex intermediate (C_i_) suggests early binding of exon-ligation factors before Prp16 action. The previously observed C conformation may be in equilibrium with our novel C_i_ conformation.

Following assembly of the spliceosome (Wilkinson et al., 2020), branching occurs in the B^∗^ complex when the 2′-hydroxyl of the brA attacks the 5′-SS (Figure 1A). The resulting C complex is remodeled by the DEAH-box ATPase Prp16 into the C^∗^ open conformation (Figure 1B). Docking of the 3′-splice site (3′-SS) at the active site (Fica et al., 2017; Wilkinson et al., 2017) forms the closed C^∗^ complex, which then catalyzes exon ligation, when the 3′-hydroxyl of the 5′-exon attacks the 3′-SS, resulting in mRNA formation and excision of the lariat-intron (Figures 1A and 1B). Exon ligation forms the post-catalytic P complex (Figure 1B), from which the ATPase Prp22 releases the mRNA.

Step-specific splicing factors associate with the spliceosome during branching and exon ligation to promote splicing chemistry. In budding yeast (*Saccharomyces cerevisiae*) the branching factors Cwc25, Yju2, and Isy1 (Chiu et al., 2009; Liu et al., 2007b; Villa and Guthrie, 2005; Warkocki et al., 2009) clamp the branch helix and dock it at the spliceosome active site (Galej et al., 2016; Wan et al., 2016, 2019). The exon-ligation factors Prp18, Slu7, and Prp17 act more indirectly. Prp18 inserts a conserved loop near the active site to stabilize the docked 3′-SS. Slu7 binds the spliceosome surface and rigidifies the C^∗^ conformation, while also promoting chemistry for substrates with a long distance between the brA and the 3′-SS (Bai et al., 2017; Brys and Schwer, 1996; Fica et al., 2017; James et al., 2002; Liu et al., 2017; Wilkinson et al., 2017; Yan et al., 2017). Prp17 binds the spliceosome before branching (Haselbach et al., 2018) but functions during exon ligation (Jones et al., 1995) to stabilize the C^∗^ complex (Fica et al., 2017; Wilkinson et al., 2017).

While a single active site catalyzes both splicing steps (Fica et al., 2013; Galej et al., 2016; Wilkinson et al., 2017), a structural rearrangement allows the 3′-SS to replace the branch helix in the active site during exon ligation (Fica et al., 2017; Wilkinson et al., 2017). Genetic experiments have suggested a two-state model of the catalytic spliceosome, in which the branching and exon-ligation conformations exist in equilibrium (Query and Konarska, 2004). This equilibrium is modulated by Prp8 and the ATPase Prp16 (Liu et al., 2007a; Tseng et al., 2011), whose activity promotes exon ligation in the forward direction by dissociating branching factors and undocking the branch helix from the active site (Figures 1B and 1E) (Semlow et al., 2016; Wilkinson et al., 2020). Both catalytic steps of splicing are reversible *in vitro*, and disrupting the function of step-specific factors promotes reversal (Tseng et al., 2017; Tseng and Cheng, 2008). Intriguingly, the exon-ligation factors Slu7 and Prp18 have been suggested to bind to weak affinity sites already in B^∗^, while Prp16 action would convert these binding sites to higher-affinity sites in C^∗^ (Ohrt et al., 2013). Slu7 and Prp18 have also been detected by mass spectrometry in some previous purifications of the C complex (Fabrizio et al., 2009). Nonetheless, it remains unclear whether step-specific factors bind exclusively to either the branching or the exon-ligation conformation of the spliceosome.

We sought to determine the molecular basis for conformational equilibrium of the catalytic spliceosome by investigating whether branching and exon-ligation factors can engage the C-complex conformation at the same time. We present a detailed analysis of a large electron cryomicroscopy (cryo-EM) dataset of the C-complex spliceosome from budding yeast. The new cryo-EM reconstruction of the C complex at 2.8 Å resolution allowed building of the most complete atomic model of a catalytic spliceosome (Figures 1C and 1D). The RNA-based active site is stabilized by both monovalent and divalent cations and uses non-Watson-Crick base pairs for splice site recognition. Most important, focused classification revealed a new C-complex intermediate (C_i_) (Figure 1E), which pre-recruits the exon-ligation factors Prp18 and Slu7. Biochemical assays suggest that these pre-bound factors are competent to promote exon ligation upon conversion to the C^∗^ conformation by Prp16. Finally, a complete model of the NTC shows how Syf1 acts as a recruitment hub for the step-specific factors Yju2 and Isy1, whose binding is remodeled in the transition from branching to exon ligation. Thus, conformational equilibrium of the catalytic spliceosome is mediated by dynamic binding of step-specific factors to the C or C^∗^ conformation.

## Results

### The complete structure of the yeast C-complex spliceosome

We previously reported a 3.8-Å structure of the C-complex spliceosome stalled with a 3′-SS mutation and purified by affinity tags on Prp18 and Slu7 (Galej et al., 2016). While the 3′-SS UAc mutation was expected to stall spliceosomes in the C^∗^ conformation (Fabrizio et al., 2009; Schwer and Guthrie, 1992), accumulation of C complexes suggested that in the absence of stable 3′-SS docking, the spliceosome equilibrates back into the branching conformation (Figure 1B) without complete dissociation of exon-ligation factors. Stalling the C^∗^ complex using a chemical modification of the 3′-SS also produced significant amounts of spliceosomes in the C conformation, which remained associated with Slu7 (Fica et al., 2017). These observations raised the possibility that exon-ligation factors may bind the C complex before Prp16 remodeling.

We merged all C-complex particles obtained by cryo-EM from various spliceosome purifications and investigated binding of Slu7 and Prp18 by focused classification (Figures S1–S4; Tables 1 and S1; Data S1). This yielded a cryo-EM reconstruction at 2.8 Å resolution for the C-complex core, showing complete base separation and base and side-chain rotamers and allowing discrimination between adenosine and guanosine bases (Figure S5). This map allowed high-confidence modeling of every protein and RNA in the core of the catalytic spliceosome. The large particle number facilitated classification and focused refinement for the peripheral flexible regions of the spliceosome (Figures S2 and S3). This produced improved maps for the U2 small nuclear ribonucleoprotein (snRNP) (3.6 Å resolution) and NTC (3.8 Å resolution), and allowed atomic modeling of these regions, which in all previous yeast spliceosome structures were modeled only by homology. The C-complex helicase module (Brr2 and Prp16) and Prp19 module (Prp19, Snt309, Cef1 C terminus) were refined to sub-nanometer resolution (6–7 Å), allowing discrimination of secondary structures and model building by molecular dynamics flexible fitting of crystal structures and homology models (Figures S2–S5). Together, these improved maps allow us to present the most complete molecular model of a catalytic spliceosome (Figures 1C and 1D; Table S1). The structure reveals novel features of the active site and unexpected binding of Prp18 and Slu7 to C complex, thus shedding light on remodeling during catalysis.

**Table 1:** Cryo-EM data processing, refinement, and validation statistics, related to Figure 1

Map and model	C-complex core	C_i_-complex composite
**Data processing**		

No. particle images in final reconstruction	403,474	See Table S1
Map resolution^a^ (FSC = 0.143) (Å)	2.80	Composite map
Map resolution range^b^ (Å)	2.71–4.94	2.85–10.6
Map resolution after density modification (Å)	2.69	See Table S1

**Refinement**		All

Model resolution (FSC = 0.5) (Å)	2.79	3.1
Map CC (around atoms)	0.70	See Table S1
Map sharpening *B* factor (Å^2^)	−89	See Table S1
Model composition		
Non-hydrogen atoms	51,675	109,106
Protein residues	5,463	12,316
RNA residues	342	545
Ligands	17	17
Mean *B* factors (Å^2^)		
Protein	100.3	134.09
RNA	129.7	156.50
Ligand	104.8	104.41
Root mean square deviations		
Bond lengths (Å)	0.009	0.010
Bond angles (°)	1.392	1.547
Validation		
MolProbity score	0.54	0.71
Clashscore	0.10	0.25
Rotamer outliers (%)	0.08	0.17
CaBLAM outliers (%)	0.76	0.98
RNA geometry (%)		
Correct sugar puckers	95.0	95.0
Good backbone conformations	80.1	78.5
Ramachandran plot (%)		
Favored	98.40	97.44
Allowed	1.59	2.51
Disallowed	0.02	0.05

**Data deposition**		

EMDB (map)	EMD-12107	EMD-12106
PDB (model)	7B9V	7B9V

The core and composite maps and models were obtained by merging 9 datasets, each with specific data collection parameters. For details of data collection for individual datasets see STAR methods, Figures S2 and S3, and references therein.

a FSC, Fourier Shell Correlation.

b Range from 5^th^ to 95^th^ percentiles of local resolution map within the refinement mask.

### Monovalent metal ion binding in the spliceosome active site

**Figure 2:**
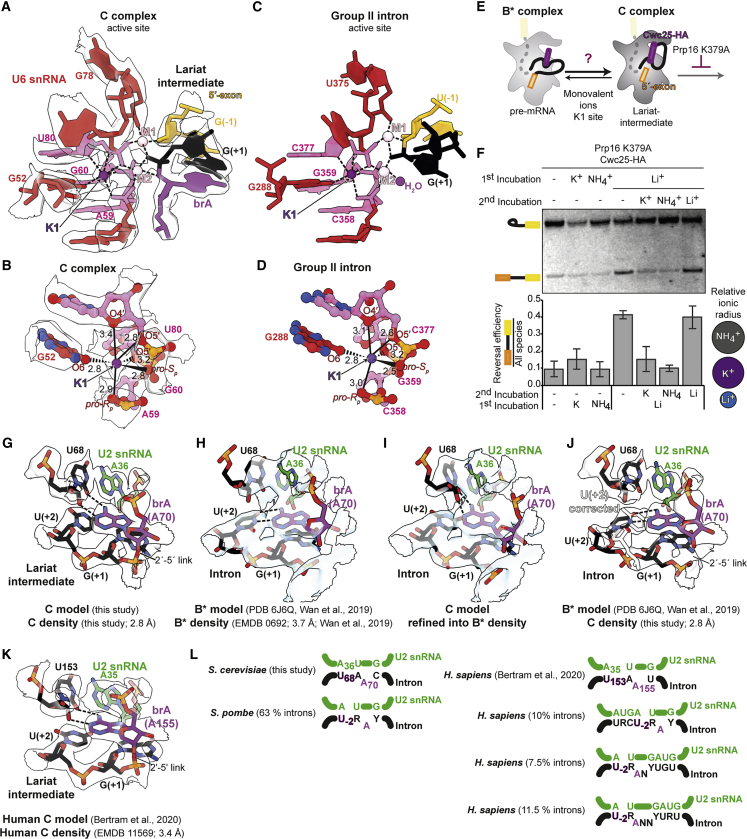
Key active site elements of the C complex (A) A potassium (K^+^) ion stabilizes the active site of the spliceosome. Metal coordination is shown as dashed lines; cryo-EM density is shown as transparent contour surface. (B) Details of K1 coordination in the spliceosome. Distances are labeled in angstroms. (C) Structure of the M1-M2-K1 catalytic metal cluster in a group IIC intron before hydrolysis (PDB: 4FAQ) (Marcia and Pyle, 2012). (D) Details of K1 coordination in the group II intron. Distances are labeled in angstroms. (E) Experimental scheme to assess the requirement for a monovalent ion during branching. (F) A monovalent ion with the ionic radius of K^+^ promotes branching. The substrate was 3′ end labeled with Cy2 before spliceosome assembly. Reversal is quantified below the gel; values are averages of 4 independent experiments; error bars represent ± standard deviation (n = 4). (G–J) Recognition of the branch adenosine (brA) during branching in yeast. Comparison of our proposed brA pairing in B^∗^ and C with the previously suggested brA interactions in B^∗^. (K) Recognition of the brA during branching in humans. (L) The pairing mode around the brA is conserved across evolution. Examples of pairing from *Schizosaccharomyces pombe* and *Homo sapiens* were derived from bioinformatic analysis in Taggart et al. (2017).

The improved resolution of our new C-complex map allows a finer analysis of the active site of the spliceosome. The active site is formed by the U6 snRNA, which adopts a triplex conformation to position two catalytic Mg^2+^ ions during branching and exon ligation (Fica et al., 2013, 2014; Steitz and Steitz, 1993; Wilkinson et al., 2020). The spliceosome uses the same active site configuration and the same two-metal ion mechanism for catalysis as observed in group II self-splicing introns (Fica et al., 2013; Marcia and Pyle, 2012), and recent structural studies support evolution of the spliceosome from group II introns (Galej et al., 2018; Haack and Toor, 2020). The active site of group II introns binds a composite, trinuclear metal cluster, in which the two catalytic Mg^2+^ ions (M1 and M2) are stabilized by a third monovalent ion, usually K^+^ (K1; Figure 2) (Marcia and Pyle, 2012; Robart et al., 2014), which promotes efficient branching by some group II introns (Marcia and Pyle, 2012). Thus, an additional positive charge may be required to stabilize M1 and M2 binding during spliceosome catalysis. Non-catalytic Mg^2+^ ions were suggested to stabilize the U6 snRNA triplex in several spliceosome structures (Wan et al., 2019; Yan et al., 2016), yet no unambiguous density for K1 had been observed so far, despite several studies suggesting that pre-mRNA splicing requires monovalent cations (Hardy et al., 1984; Lin et al., 1985).

In our 2.8-Å C-complex map, we observed a new spherical density clearly separated from adjacent phosphates and bases in a position corresponding to K1 in group II introns (Figure 2A). This density has octahedral coordination geometry from surrounding oxygen ligands, with bond distances of 2.7–3 Å, consistent with a monovalent cation (Auffinger et al., 2011) (Figures 2A and 2B). As our spliceosomes were purified using buffers containing K^+^, we assigned this density as a K^+^ ion. This ion interacts with 3 of the 4 U6 snRNA residues that provide ligands for the catalytic Mg^2+^ ions, including the ligands for M2 (A59, G60, and U80), as well as with the third triplex strand (G52), and is thus ideally positioned to stabilize M1 and M2 binding in the active site, as does K1 in group II introns (Figures 2C and 2D). Supporting our assignment, the K1 site is not occupied when yeast or human C complexes are purified in buffers containing Na^+^ (Wan et al., 2016; Bertram et al., 2020) (Figure S6N), consistent with the shorter coordination distance observed for Na^+^, which would not allow Na^+^ to bind stably at this site (Auffinger et al., 2011).

To test whether a monovalent ion with the ionic radius of K^+^ promotes splicing, we took advantage of the reversibility of the branching reaction to investigate whether monovalent ions could modulate the forward and reverse reactions in C complex, as suggested previously (Tseng and Cheng, 2008, 2013). We stalled C complexes with a Prp16 mutant and purified them with Cwc25 to remove K^+^ normally present in *in vitro* splicing reactions (Figure 2E). Stalled C complexes could catalyze reverse branching efficiently to make pre-mRNA from lariat-intermediate in the presence of Li^+^, which has a much smaller ionic radius than K^+^, but only very poorly in K^+^ or NH_4_^+^, which has a ionic radius similar to that of K^+^ (Figure 2F) (Shannon, 1976). Thus, a monovalent ion with the ionic radius of K promotes the forward reaction during branching in C complex. To test this conclusion further, we asked whether K^+^ could promote the conversion of pre-mRNA to lariat-intermediate from the B^∗^ state. We reverted spliceosomes to the B^∗^ state in Li^+^. After washing away Li^+^, these complexes could catalyze branching when K^+^ or NH_4_^+^ was added back, whereas Li^+^ prevented branching of the pre-mRNA into lariat-intermediate (Figure 2F). Thus, a monovalent metal ion modulates equilibrium between forward and reverse reactions during branching. These functional studies support our assignment of K^+^ binding at the K1 site. Since branching can reverse in the absence of K^+^, our results suggest that binding of K^+^ at K1 may also modulate the equilibrium between branching and exon ligation, as does the analogous ion site in the group II intron (Manigrasso et al., 2020; Marcia and Pyle, 2012).

Intriguingly, right after the formation of the U6 snRNA triplex, in B^act^, the K1 site is partially blocked by a lysine from Prp11, a U2 snRNP protein that is dissociated by Prp2 during catalytic activation (Yan et al., 2016) (Figure S6A). In the late B^∗^ complex, before docking of brA at the active site, the K1 site remains unoccupied (Wan et al., 2019). The K1 site is fully occupied for the first time only in C complex and this site remains filled in P complex cryo-EM maps, provided that K^+^ is present in the purification buffers (Liu et al., 2017) (Figures S6B–S6L). Such progressive occupancy of the K1 site is conserved in human spliceosomes (Haselbach et al., 2018) (Figures S6M–S6O). We propose that formation of the full M1-M2-K1 metal cluster is necessary for catalytic competence and may serve to couple docking of the brA at the active site to catalytic activation of the spliceosome. This metal cluster is maintained throughout the catalytic stage and promotes both branching and exon ligation, similarly to its group II intron equivalent (Marcia and Pyle, 2012).

### Non-Watson-Crick base pairs recognize the branch adenosine during branching

The brA bulges out of the branch helix formed between the intron and U2 snRNA and must be precisely positioned for nucleophilic attack on the 5′-SS (Galej et al., 2016; Wilkinson et al., 2020). Recognition of the brA is thus essential for efficient branching, and specific mutations of this adenosine inhibit branching *in vitro* and *in vivo* (Lesser and Guthrie, 1993; Vijayraghavan et al., 1986) during the catalytic stage (Tseng et al., 2011) by destabilizing the branching conformation (Query and Konarska, 2004). In our original 3.8 Å structure of C complex (Galej et al., 2016), the brA was built with its Watson-Crick face paired with the sugar edge of intron nucleotide U(+68), two nucleotides upstream (UACUA**A**C—brA in bold, U(+68) underlined). However, in a different structure of C complex at 3.4 Å (Wan et al., 2016) this pairing was not modeled. Instead, in subsequent structures of a B^∗^ complex poised for branching (Wan et al., 2019), a canonical Watson-Crick base pair was proposed between brA and the 5′-SS nucleotide U(+2), although in these structures, the local resolution around the brA was limited (∼4 Å) (Figure 2H). Therefore, the structural basis for the nucleotide specificity of the branching reaction was unclear.

In our new structure of C complex, the branch helix is bound by Yju2 and Isy1 and is visible at a local resolution of 2.8 Å around the brA. The density unambiguously demonstrates that brA pairs to the sugar edge of U(+68) in a base-triple interaction (Figure 2G), while the 5′-SS U(+2) pairs with U2 snRNA G37 in the context of another base triple, as we proposed previously (Galej et al., 2016). Our model can be convincingly refined into density for the B^∗^ complex poised for branching (Figures 2H and 2I), in which Yju2 and Isy1 are bound to the branch helix (Wan et al., 2019). The previous, incorrect model of this interaction in B^∗^ likely originated from the ambiguity of map interpretation at ∼4 Å resolution, and there is no evidence for brA base-pair remodeling between B^∗^ and C complexes (Figures 2G–2K). Moreover, mutations at U(+2) of the intron primarily impair exon ligation, which is inconsistent with a functional role for U(+2) in pairing to brA during branching (Liu et al., 2007a, Query and Konarska, 2004). Instead, we propose that the brA is recognized for branching through non-canonical pairing with U(+68), which anchors it to the branch helix while allowing the 2′-hydroxyl to be positioned for nucleophilic attack on the 5′-SS. Our model explains the effects of specific branch adenosine mutations. Substitution of brA with cytosine or uridine strongly inhibits branching, while mutation to guanosine has milder effects *in vivo* and *in vitro* (McPheeters, 1996; Query and Konarska, 2004; Tseng et al., 2011), and these effects correlate with the level of distortion and movement of the brA 2′-OH that would result from accommodating base pairs analogous to the interaction of brA with U(+68) (Leontis et al., 2002) (Figures S6A–S6G). A similar model for pairing of brA was built in a recent cryo-EM structure of the human C complex (Bertram et al., 2020) (Figure 2K and 2L) and explains why in humans a uridine 2 nucleotides before and an adenosine at the branch are conserved in most introns (Pineda and Bradley, 2018). Genome-wide analyses of branch point usage indicate that this mode of pairing is conserved across evolution and occurs for most human introns, even when the sequence around the brA diverges from the yeast consensus (Taggart et al., 2017) (Figure 2M). These observations underscore the importance of careful interpretation of cryo-EM maps in the context of previous functional studies of the spliceosome (Mayerle and Guthrie, 2017).

### The exon-ligation factors Prp18 and Slu7 are recruited to a new on-pathway C_i_ intermediate

**Figure 3:**
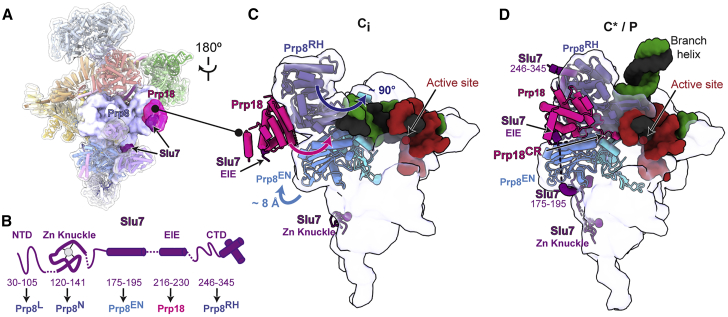
Prp18 and Slu7 are recruited to a new C_i_ conformation (A) Location of exon-ligation factors in the C-complex spliceosome. Prp8 and Prp18/Slu7 are shown as surface representation. (B) Domain architecture of Slu7 and the interaction partners of each domain. NTD, N-terminal domain; CTD, C-terminal domain. (C and D) Pre-recruited Prp18 and Slu7 are remodeled from the C_i_ to the C^∗^/P conformation. Upward movement of the Prp8 endonuclease domain (Prp8^EN^) creates additional binding sites for Slu7 in C^∗^/P. The Prp18 conserved region (CR) is disordered in C_i_ but engages the active site in C^∗^/P.

In C^∗^ and P complexes, the exon-ligation factor Prp18 binds to a face of the Prp8 RNase H domain (Prp8^RH^) that would also be accessible in C complexes (Figures 1E and 3A), suggesting that Prp18 may bind the spliceosome before Prp16-mediated remodeling. We hypothesized that such early binding of Prp18/Slu7 explained how we could purify C complexes using affinity tags on Prp18 or Slu7 (Fica et al., 2017; Galej et al., 2016). Using focused classification, we identified a subset of particles in the C-complex conformation with extra density on the same face of Prp8^RH^ that Prp18 binds in C^∗^ (Figures 1D, 1E, 3C, S3B, and S7). The α-helical domain of Prp18 visible in C^∗^ was a perfect fit for this density, which also accommodated a small peptide of Slu7 (termed the EIE element; Figures 3B, 3C, and S7A) that is known to bind Prp18 (James et al., 2002). The conserved loop of Prp18 that stabilizes the 3′-SS docked in the active site in C^∗^/P is not visible in this subset of particles (Prp18^CR^; Wilkinson et al., 2017; Figures 3C and 3D), suggesting it is disordered before Prp16 remodeling and 3′-SS docking. Biochemical experiments suggest that endogenous Prp18 and Slu7 form a heterodimer (James et al., 2002; Zhang and Schwer, 1997), and thus early binding of Prp18 would also imply early binding of Slu7. We also observed density corresponding to the Zn knuckle of Slu7 (ZnK) bound to Prp8 in this new intermediate (Figures 1E, 3B, 3C, and S7). This novel intermediate between C and C^∗^, which we called C_i_, demonstrates that all branching and exon-ligation factors can be bound in one complex, in agreement with previous biochemistry (Ohrt et al., 2013).

**Figure 4:**
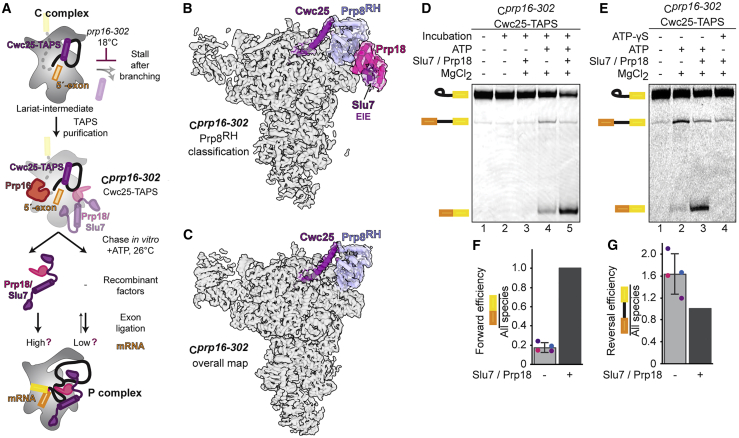
The novel C_i_ conformation is an on-pathway intermediate (A) Biochemical assay to assess function of the C_i_ conformation. *Prp16-302* impairs ATP hydrolysis at low temperatures and stalls Prp16 on the spliceosome right after branching. Chase of the purified C^*prp16-302*^ complex at the permissive temperature is predicted to allow mRNA production, even in the absence of recombinant exon-ligation factors. (B and C) Focused classification reveals a subpopulation of C^*prp16-302*^ spliceosomes in the C_i_ conformation. Maps were reconstructed without refinement from the classified particles and filtered to 5 Å to aid comparison of additional density for Prp18/Slu7. Prp8^RH^, Prp8 RNase H domain. (D) Purified C^*prp16-302*^ complexes produce mRNA when chased in the presence of ATP. The substrate was 3′ end labeled with Cy2 before spliceosome assembly. (E) ATP hydrolysis is required for both exon ligation and branching reversal by C^*prp16-302*^ spliceosomes. (F and G) Quantification of exon ligation and branching reversal efficiency for C^*prp16-302*^ spliceosomes. Relative ratios were normalized in each experiment to the condition in which recombinant Slu7/Prp18 was added. Values are averages of 4 independent experiments from 3 independent spliceosome purifications; error bars represent ± standard deviation (n = 4). Values for individual experiments are indicated as dots overlaid on the bar graphs.

To address the functional relevance of early binding of Prp18/Slu7 before Prp16 remodeling, we investigated whether the C_i_ intermediate could be purified independently of Prp18 and Slu7 using a branching factor that binds stably to the C complex before Prp16 action. We assembled native complexes on wild-type pre-mRNA and stalled the C complex with a cold-sensitive Prp16 mutation (*prp16-302*) that impairs ATP hydrolysis (C^*prp16-302*^; Figure 4A). We purified the stalled C-complex spliceosomes with the branching factor Cwc25, which remains bound after branching when Prp16 action is blocked (Tseng et al., 2017; Warkocki et al., 2009). Cryo-EM reconstruction revealed that these C complexes were structurally identical to the C complex assembled on the 3′-SS UAc substrate (Figures 4B, 4C, S3, S7B, and S7C). Focused classification identified a subset of particles in the C_i_ conformation, in which Cwc25 and Prp18/Slu7 were bound to the same complex (Figures 4B, S3, and S7D), and we could identify Slu7 and Prp18 by mass spectrometry in the purified C^*prp16-302*^ sample (Figure S1A). Thus, Prp18 and Slu7 can bind to the spliceosome before Prp16 action, and the C_i_ complex can be purified with a branching factor.

Overall, an average of 30% of C complexes resulting from various stalls during catalysis are in the C_i_ conformation and contain Prp18 and Slu7 (Figure S7). However, only the small EIE element of Slu7 that interacts with Prp18 is present in all C_i_ particles, while the ZnK of Slu7 engages only a subpopulation of C_i_ complexes (Figures 1D, 1E, and S7). The binding of Prp18 and of the Slu7 ZnK can occur independently of each other, but focused classification indicated that the binding of Prp18 increases the odds for the binding of the Slu7 ZnK, and vice versa (odds ratio = 1.35). This relationship was observed regardless of the stall used to obtain the C_i_ intermediate, providing evidence that Prp18 and Slu7 interact as a heterodimer independently of their binding to the spliceosome (Figure S7), in agreement with previous biochemistry (James et al., 2002). This mode of binding may allow the early recruitment of Prp18 and/or Slu7 while ensuring that stable engagement of both exon-ligation factors with the spliceosome occurs only after Prp16 remodeling.

To determine whether C_i_ is a functional on-pathway intermediate, we assayed whether C^*prp16-302*^ complexes that contain a subpopulation of spliceosomes in the C_i_ conformation could produce mRNA without the addition of exogenous exon-ligation factors. We chased purified C^*prp16-302*^ complexes at the permissive temperature, as the *prp16-302* mutation allows growth, and therefore splicing *in vivo* at 25°C (Madhani and Guthrie, 1994). Incubation of C^*prp16-302*^ complexes at 25°C without ATP or with non-hydrolyzable ATP-γS did not allow exon ligation, demonstrating that these complexes are stalled before Prp16 has acted to allow remodeling to C^∗^ (Figures 4D and 4E) (Schwer and Guthrie, 1992). Incubation with ATP and exogenous, recombinant Prp18/Slu7 resulted in efficient exon ligation, showing the spliceosomes are on-pathway intermediates. Importantly, incubation with ATP in the absence of exogenous Prp18/Slu7 still produced significant levels of mRNA (Figures 4D–4F), demonstrating that the small C_i_ population containing Prp18/Slu7 (Figure S7D) stalled by the *prp16-302* mutation represents a functional on-pathway intermediate. Since Slu7/Prp18 are necessary for UBC4 splicing (Semlow et al., 2016), we infer that Prp18/Slu7 pre-bound in C_i_ are competent to promote exon ligation upon remodeling by Prp16. Intriguingly, in the absence of exogenous Prp18/Slu7, C^*prp16-302*^ complexes also catalyzed reverse branching to make pre-mRNA (Figures 4D–4G). Thus, when Prp18 and Slu7 are limiting, the C_i_ spliceosome can revert to the B^∗^ conformation, supporting the idea that Prp18 and Slu7 shift the equilibrium toward the C^∗^ conformation during remodeling (Fica et al., 2017; Semlow et al., 2016; Wilkinson et al., 2017).

### Complete structure of the NTC reveals basis for recruitment of branching factors

**Figure 5:**
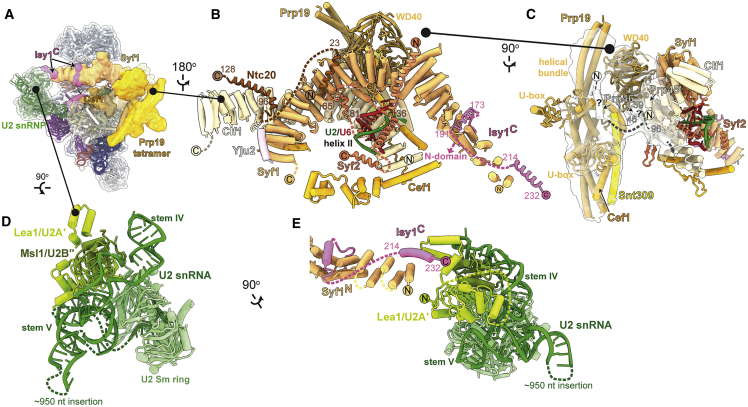
Complete architecture of the NTC and U2 snRNP in C complex (A) Location of NTC and U2snRNP in C complex. (B) Architecture of the NTC showing newly identified Ntc20, Syf2, and Isy1 elements. (C) The N-termini of Prp45 and Prp46 mediate attachment of the Prp19 helical bundle to the spliceosome. Focus-classified Prp19 density is shown as a transparent contour surface. (D) Complete structure of the U2 snRNP in C complex. *S. cerevisiae* contains a large, flexible insertion in U2 snRNA that is not visible in our map. (E) Isy1 bridges the NTC to U2 snRNP through Lea1.

The NTC is one of the biggest pre-assembled components of the spliceosome and joins during formation of the active site triplex in the B^act^ complex (Plaschka et al., 2019; Wilkinson et al., 2020). The NTC stabilizes the U6 triplex (Fica et al., 2014) and is essential for correct docking of the 5′-SS in the active site (Chan and Cheng, 2005; Chan et al., 2003). Moreover, branching factors were initially identified as proteins that interact loosely with the NTC, suggesting the NTC could influence conformational equilibrium between branching and exon-ligation (Chang et al., 2009; Chen et al., 2001; Chiu et al., 2009; Liu et al., 2007b; Villa and Guthrie, 2005). Previous yeast spliceosome structures revealed the overall architecture of the NTC as a sprawling clamp centered on a hinge formed by the tetrameric helical bundle of Prp19, from which Syf1 and Clf1 emerge as two helical arches that allow other components such as Cef1 to engage the spliceosome core (Figure 5A). The Syf1 and Clf1 arches are very mobile during the C to C^∗^ transition (Plaschka et al., 2019; Wilkinson et al., 2020), and this feature has reduced local resolution for these regions in previous spliceosome maps. Thus, most of Clf1 and Syf1 were previously built only as idealized alpha helices of uncertain register.

Our large C-complex dataset allowed focused refinement that significantly improved density quality for Syf1 and Clf1 (Figure S3), showing surprisingly that several of the alpha helices previously assigned to Syf1 come from NTC proteins Syf2, Isy1, and Ntc20 and the U2 snRNP protein Lea1. We were able to build the N-terminal domain (NTD) of Syf2 for the first time, showing how Syf2 bridges Syf1 and U2/U6 helix II to anchor the NTC to active site elements in the core through Cef1 (Figure 5B). Three helices on the surface of Clf1 were assigned to the N-termini of Prp45 and Prp46, with the latter projecting toward the Prp19 helical bundle, possibly acting as a tether for this domain (Figure 5C). We also identified Ntc20, the only remaining unassigned NTC component. Two helices from Ntc20 bind the C-terminal domains (CTDs) of Syf1 and Clf1, respectively (Figures 5B and S5), thus linking these arches during activation and catalysis, which is consistent with previous genetics and biochemistry (Chang et al., 2009; Chen et al., 2001).

Our new map also explains how Isy1 (also known as Ntc30) is recruited to the spliceosome to act as a branching factor. The NTD of Isy1 was previously built in C complex as a clamp element that promotes docking of the branch helix at the active site (Galej et al., 2016; Wan et al., 2016). We identified two helices from the C terminus of Isy1 that bind the N terminus of Syf1 (Figure 5B), explaining previous biochemical and genetic data implicating Isy1 as a peripheral NTC component (Chen et al., 2001). Thus, the Isy1 NTD, which promotes branching, is likely already bound in B^act^ and tethered close to its site of action after Prp2 activity, in a manner parallel to Prp18 binding before exon ligation in C_i_. Our improved model of the U2 snRNP (Figures 5A and 5D) shows that the C-terminal helix of Isy1 also bridges the extreme N terminus of Syf1 to a newly identified 3-helix bundle of the U2 snRNP component Lea1 (Figures 5D and 5E). Thus, Isy1 acts as a link between the NTC and the U2 snRNP, and this interaction may be maintained throughout the catalytic stage and may influence C to C^∗^ remodeling, although Isy1 has yet to be identified in maps of C^∗^ or P complexes.

## Discussion

**Figure 6:**
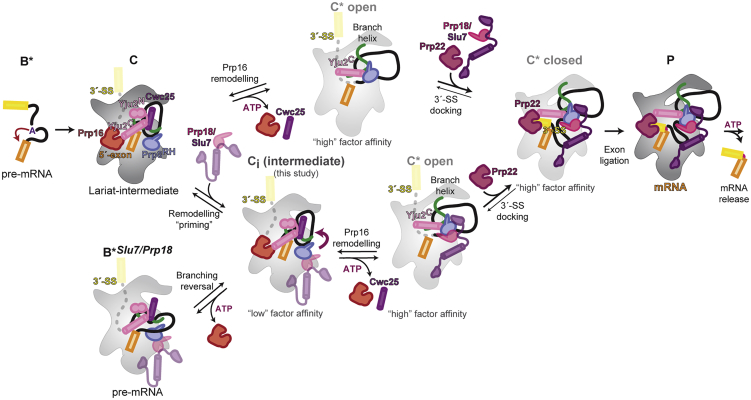
Model for conformational equilibrium during the catalytic stage of pre-mRNA splicing Following branching, the exon-ligation factors Slu7 and Prp18 can bind the C conformation with low affinity by interacting with the Prp8^RH^ domain to prime remodeling, thus forming the C_i_ intermediate. Prp16-mediated remodeling of the C_i_ complex leads to a C^∗^ “open” conformation, with high affinity for Slu7/Prp18. Dissociation of Cwc25 from C_i_ allows the Yju2 C-terminal domain (Yju2^C^) to interact with Prp8^RH^ and stabilize the binding of Prp22. The undocked branch helix is locked in a C^∗^ “closed” conformation by the stable binding of Prp18 and Slu7 and by Yju2^C^. The C^∗^ closed conformation allows stable 3′-SS docking and exon ligation. The C_i_ complex is likely unstable, and during Prp16 activity, in the absence of additional Slu7/ Prp18 binding, remodeling can also allow C_i_ to catalyze the reversal of branching, thus reverting to a B^∗^ conformation in which Slu7/Prp18 may remain bound (B^∗*Slu7/Prp18*^). Remodeling of the C complex may also occur through a pathway that does not involve C_i_, where Prp16 action precedes or is concomitant with the recruitment of Slu7/Prp18 in the C^∗^ open conformation.

Our high-resolution structure of the C complex shows how the single active site of the spliceosome positions a trinuclear Mg^2+^/K^+^ cluster for catalysis (Figure 2). The active site remains catalytically licensed from B^∗^ onward (Figure S6), but juxtaposes different reactants for each catalytic step. Although the 5′-exon remains bound to U5 snRNA for both steps, the branch helix and brA are removed from the active site after branching to make space for the 3′-SS to dock near the 5′-exon during exon ligation (Wilkinson et al., 2020). The ATPase Prp16 drives the remodeling of brA interactions after branching to allow brA pairing to the 3′-SS, while Prp8 cradles the active site and governs a dynamic equilibrium between the branching and exon-ligation conformations (Fica and Nagai, 2017; Liu et al., 2007a; Query and Konarska, 2004). Step-specific factors modulate this equilibrium (Ohrt et al., 2013; Tseng et al., 2017; Tseng and Cheng, 2008; Warkocki et al., 2009; Wilkinson et al., 2020). The branching factors Cwc25 and Isy1 dissociate from the C-complex active site to allow the exchange of reactants in the active site in C^∗^/P (Wilkinson et al., 2020), while the exon-ligation factors Slu7 and Prp18 stabilize the C^∗^/P conformation to promote docking of the 3′-SS (Wilkinson et al., 2020). Unexpectedly, we found that ∼30% of C complex particles that retained branching factors (e.g., Cwc25) also contained the exon-ligation factor Prp18 (Figure S7), demonstrating that exon-ligation factors can bind the branching conformation before Prp16 remodeling. This new C_i_ intermediate, in which branching and exon-ligation factors bind the same complex (Figures 1E and 6), provides the structural basis for how the spliceosome toggles between the branching and exon-ligation conformations.

In C_i_, Prp18 binds the Prp8^RH^ domain before Prp16 remodeling, and potentially before branching catalysis (Ohrt et al., 2013). Slu7 is recruited to C_i_ through interactions of its EIE element with Prp18 or through binding of its ZnK domain to the NTD of Prp8 (Figure 6). Binding of the remainder of Slu7 is prevented in C_i_ by Cwc25, which would clash with the CTD of Slu7, while engagement of the central region of Slu7 requires upward movement of the Prp8^EN^ domain in C^∗^ (Figure 3). Our C_i_ structure explains why previous studies have observed binding of Slu7 and Prp18 before Prp16 action at low salt concentrations and thus proposed a weaker affinity site before remodeling by Prp16 (Ohrt et al., 2013). In fact, our data suggest that exon-ligation factors bind at the same sites before and after Prp16 action, but their affinity likely increases following remodeling by Prp16 (Figure 6).

Consistently, at least 30% of C complexes stalled with the conditional *prp16-302* allele and purified at low salt concentrations contain Prp18 in the C_i_ state (Figure S7). These complexes can be reactivated to produce mRNA upon ATP hydrolysis by Prp16, even in the absence of exogenous Slu7 and Prp18 added following purification (Figure 4). C_i_ is thus a key on-pathway intermediate in remodeling between branching and exon ligation (Figure 6); following ATP hydrolysis by Prp16, Prp18 and Slu7 pre-bound in C_i_ can stably engage the spliceosome to stabilize the higher-energy C^∗^ complex and promote exon ligation (Figures 4D–4G and 6).

**Figure 7:**
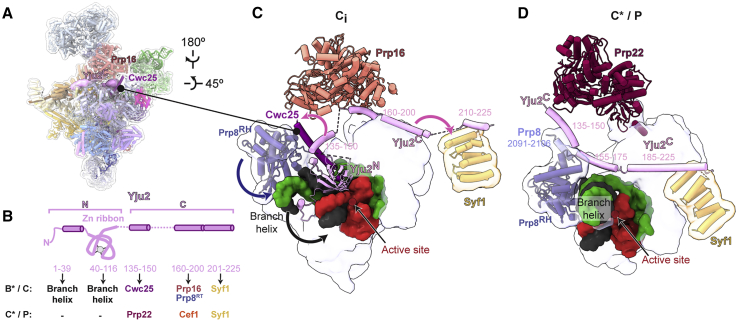
Remodeling of Yju2 interactions during the C to C^∗^ transition (A) Location of the branching factor Yju2 in the C-complex spliceosome. (B) Domain architecture of Yju2 and its interaction partners during branching and exon ligation. (C) Yju2 supports binding of Cwc25 and Prp16 in C complex. (D) Yju2 is remodeled in C^∗^/P to act as exon-ligation factor. Dissociation of Cwc25 is required for formation of a new Prp8 helix that supports Yju2-mediated recruitment of Prp22 in C^∗^/P.

The binding of branching factors is also remodeled during Prp16 activity. In B^∗^/C complexes, branching is promoted by engagement of the N-termini of Cwc25, Yju2, and Isy1 with the branch helix and brA, while the C termini of these branching factors interact with the NTC and Prp16. Two C-terminal helices of Yju2 cross Cwc25 before interacting with the NTC, forming a unique binding platform for Prp16 at the branching stage (Wan et al., 2019; Zhang et al., 2017) (Figures 7A–7C). These Yju2 helices are reorganized in C^∗^/P complexes to bridge the rotated Prp8^RH^ domain to Prp22, while the Yju2 N-terminal domain dissociates (Figure 7D). While the N terminus of Yju2 is essential for viability and promotes branching, deletion of the C terminus allows only inefficient exon ligation in the absence of Prp16 (Chiang and Cheng, 2013), suggesting that Yju2 stabilizes both C and C^∗^ in a manner consistent with our structural model (Figures 6 and 7). We further show that the C terminus of Isy1 also binds the NTC, gluing the interface between Syf1 and the U2 snRNP (Figure 5). Although detailed structures of the NTC are not yet available in the B^act^ and C^∗^/P complexes, this interaction is likely preserved after remodeling to the C^∗^ complex, while the Isy1 N-terminal domain similarly dissociates. Thus, paralleling the primed association of exon-ligation factors with the branching conformation in C_i_ complex, the branching factors Isy1 and Yju2 are both associated with the exon-ligation conformation in C^∗^ and P complexes. Such tethering of Isy1 by the NTC throughout catalysis explains how Isy1 affects proofreading of both the branch site and 3′-SS (Villa and Guthrie, 2005).

The C_i_ intermediate thus shows that complexes in the branching conformation (B^∗^/C/C_i_) differ little in composition from those in the exon-ligation conformation (C^∗^/P). Only Cwc25 dissociates from branching to exon ligation, while all of the other factors can already bind in the C conformation and are remodeled as a result of Prp16 action (Figure 6). Cwc25 locks the spliceosome in the branching conformation and prevents both the forward pathway, to exon ligation, and the reversal of branching (Tseng et al., 2017). Consequently, the C conformation is a particularly stable state, as complexes that cannot complete catalysis appear to revert to this state. C complexes accumulate not only when Prp16 remodeling is blocked but also when exon ligation is impaired by mutation of the 3′-SS, or when mRNA release is prevented by blocking Prp22 activity (Figure S2). Therefore, the energy of ATP hydrolysis by Prp16 is necessary to disrupt this stable conformation and establish an equilibrium between branching and exon ligation that underlies the reversibility of both splicing reactions (Tseng and Cheng, 2008).

The C_i_ spliceosome shows how exon-ligation factors can prime remodeling before Prp16 activity and shift the equilibrium from branching to exon ligation after Prp16 hydrolyzes ATP. By stabilizing the higher-energy C^∗^ conformation, exon-ligation factors act like a Brownian ratchet, similar to how branching factors drive the equilibrium toward B^∗^ during spliceosome activation by Prp2 (Krishnan et al., 2013; cf. Semlow et al., 2016). Specific branching factors further modulate this equilibrium through the partial dissociation of their NTDs as a result of Prp16 action (Figure 6). Conversely, when Slu7 and Prp18 are limiting, the spliceosome cannot be “locked” into the exon-ligation conformation, and Prp16 action allows the spliceosome to reverse branch and revert to the B^∗^ conformation (Figures 4D–4G and 6). Such reversibility may be important during the proofreading of branching by Prp16 (Burgess and Guthrie, 1993; Semlow et al., 2016) and suggests that Slu7 and Prp18 may affect catalysis and proofreading more broadly, by modulating the equilibrium between B^∗^, C, C_i_, and C^∗^/P complexes. In support of this idea, we observed the C_i_ conformation when spliceosomes were stalled with a 3′-SS mutant that docks poorly in the active site but can produce mRNA when proofreading is disabled (Mayas et al., 2006; Tseng and Cheng, 2008), suggesting that the proofreading of exon ligation by Prp22 can lead to substrate rejection and collapse to the C_i_ conformation without dissociation of exon-ligation factors (Figures 6 and S7). In this context, docking of the 3′-SS in the active site to convert the C^∗^ open complex into the C^∗^ closed complex (Figure 6) is coupled to destabilization of the C_i_ conformation after ATP hydrolysis by Prp16, and drives the equilibrium forward toward exon ligation for a correct 3′-SS.

Our improved model of the catalytic stage involving the novel C_i_ conformation (Figure 6) highlights how Prp16 acts as a classical catalyst by reducing the activation barrier to transition from the very stable branching conformation to the higher-energy exon-ligation conformation. Thus, the energy of ATP hydrolysis by Prp16 allows thermodynamic control of splicing and equilibrium of the spliceosome, a feature likely necessary for the proofreading of catalysis. Genetic studies suggest that the stability of RNA elements that form the active site is also disrupted during Prp16 action in a manner that influences splice site proofreading (Eysmont et al., 2019; Fica et al., 2014).

### Limitations

Although exon-ligation factors can prime remodeling before Prp16 action, the C_i_ complex may not be an obligatory intermediate in the pathway. Instead, for some substrates, ATP hydrolysis by Prp16 may precede the binding of exon-ligation factors (Figure 6, top pathway). Supporting this possibility, the face of Prp8 bound by Prp18 in C_i_ is occluded by a human-specific protein in the structure of the human C complex assembled on an adenovirus-derived pre-mRNA (Bertram et al., 2020), suggesting that for some transcripts, Prp18 cannot engage the spliceosome before Prp16 remodeling to C^∗^.

## STAR★Methods

### Key resources table

**Table:** 

REAGENT or RESOURCE	SOURCE	IDENTIFIER
**Bacterial and virus strains**		

*Eserichia coli* BL21-CodonPlus (DE3)-RIPL	Agilent Technologies	Cat. #230280

**Chemicals, peptides, and recombinant proteins**		

Amylose Resin High Flow	NEB	Cat. #E8022S
StrepTactin Sepharose High Performance	Cytiva Europe GmbH	Cat. #28935599
Desthiobiotin	IBA GmBH	Cat. #2-1000-002
Adenosine 5′-[γ-thio]triphosphate tetralithium salt	Sigma Aldrich	Cat. #93839-89-5
Monoclonal Anti-HA−Agarose	Merck-Sigma	Cat. #A2095-1ML

**Deposited data**		

C_i_ complex core map	This paper	EMDB 12107
C_i_ complex composite map	This paper	EMDB 12106; ftp://ftp.mrc-lmb.cam.ac.uk/pub/mwilkin/Ci_spliceosome/
C_i_ complex Prp18 focused map	This paper	EMDB 12114
C_i_ complex Slu7 focused map	This paper	EMDB 12113
C complex NTC focused map	This paper	EMDB 12109
C complex U2 snRNP focused map	This paper	EMDB 12108
C complex Prp17 focused map	This paper	EMDB 12112
C complex Prp19 focused map	This paper	EMDB 12116
C complex helicase module focused map	This paper	EMDB 12110
C complex Cwc22 N terminus focused map	This paper	EMDB 12118
C complex U5 snRNA focused map	This paper	EMDB 12117
C_i_ composite model	This paper	PDB 7B9V; ftp://ftp.mrc-lmb.cam.ac.uk/pub/mwilkin/Ci_spliceosome/
*S. cerevisiae* B^∗^ complex map	Wan et al., 2019	EMDB 0692
*S. cerevisiae* B^∗^ complex model	Wan et al., 2019	PDB 6J6Q
*H. sapiens* C complex map	Bertram et al., 2020	EMDB 11569
*H. sapiens* C complex model	Bertram et al., 2020	PDB 6ZYM
*S. cerevisiae* B^act^ complex map	Yan et al., 2016	EMDB 9524
*S. cerevisiae* B^act^ complex model	Yan et al., 2016	PDB 5GM6
*S. cerevisiae* P complex map	Wilkinson et al., 2019	EMDB 10140
*S. cerevisiae* P complex model	Wilkinson et al., 2017	PDB 6EXN
*S. cerevisiae* P complex map	Liu et al., 2017	EMDB 7109
*S. cerevisiae* P complex model	Liu et al., 2017	PDB 6BK8
*H. sapiens* B^act^ complex map	Haselbach et al., 2018	EMDB 4255
*H. sapiens* B^act^ complex model	Haselbach et al., 2018	PDB 6FF4
*H. sapiens* P complex map	Fica et al., 2019	EMDB 4525
*H. sapiens* P complex model	Fica et al., 2019	PDB 6QDV

**Experimental Models: organisms/strains**		

*Saccharomyces cerevisiae prp16-302*	Fica et al., 2014	N/A

**Oligonucleotides**		

5′-SS targeting oligo: 5′-ACTTTAGACATAC-3′	Sigma Aldrich	N/A

**Recombinant DNA**		

Plasmid for Slu7/ Prp18 for expression	This paper	N/A
Plasmid for Prp16 K379A expression	This paper	N/A

**Software and algorithms**		

COOT-0.8.9.2	Casañal et al., 2020	https://www2.mrc-lmb.cam.ac.uk/personal/pemsley/coot/
PHENIX	Afonine et al., 2018	https://www.phenix-online.org/
	Terwilliger et al., 2020	
GCTF	Zhang, 2016	https://www.mrc-lmb.cam.ac.uk/kzhang/
MotionCor2	Zheng et al., 2017	https://emcore.ucsf.edu/ucsf-software
Relion 1.3 – 3.1	Scheres, 2014; Kimanius et al., 2016	https://www3.mrc-lmb.cam.ac.uk/relion/index.php?title=Main_Page
crYOLO	Wagner et al., 2019	http://sphire.mpg.de/
ISOLDE	Croll, 2018	https://isolde.cimr.cam.ac.uk/
UCSF ChimeraX	Goddard et al., 2018	http://www.cgl.ucsf.edu/chimerax/

### Resource availability

#### Lead contact

Further information and requests for resources and reagents should be directed to and will be fulfilled by the Lead Contact, Sebastian M. Fica (sfica@mrc-lmb.cam.ac.uk or sebastian.fica@bioch.ox.ac.uk)

#### Materials availability

Plasmids and yeast strains generated for this study can be obtained upon request from the Lead Contact, but we may require a payment and/or a completed Materials Transfer Agreement if there is potential for commercial application.

#### Data and code availability

The cryo-EM maps have been deposited in the Electron Microscopy Data Bank with the following accession codes: EMD-12106 (C-complex with Slu7/Prp18 bound), EMD-12107 (focused refinement on core), EMD-12108 (focused refinement on U2 snRNP), EMD-12109 (focused refinement on NTC), EMD-12110 (focused refinement on Brr2/Prp8-Jab/Prp16), EMD-12111 (focused refinement on Brr2/Prp8-Jab), EMD-12112 (overall map after focused classification on Prp17), EMD-12113 (overall map after focused classification on Slu7 Zn Knuckle), EMD-12114 (overall map after focused classification on Prp18), EMD-12115 (reconstructed signal-subtracted map after focused classification on Prp19), EMD-12116 (overall map after focused classification on Prp19), EMD-12117 (overall map after focused classification on U5 Sm ring), EMD-12118 (overall map after focused classification on Cwc22 NTD). The coordinates of the composite atomic model have been deposited in the Protein Data Bank under accession code PDB 7B9V. A PyMol session of the composite model is available online as Data S1. For immediate access, the composite map and model, as well as a PyMol session can be downloaded from the following folder, using “Connect As: Guest” if prompted for a password: ftp://ftp.mrc-lmb.cam.ac.uk/pub/mwilkin/Ci_spliceosome/

### Experimental model and subject details

#### Yeast strains

A TAPS tag cassette was added in frame to the C terminus of the endogenous CWC25 locus in *S. cerevisiae* yJPS983, carrying a chromosomal *prp16-302* allele (Fica et al., 2014), using the *kanMX6* resistance cassette (Nguyen et al., 2015). *prp16-302* encodes the *prp16R456K* in motif Ib, which significantly reduces ATP hydrolysis at low temperatures and stalls spliceosomes after branching (Lardelli et al., 2010). For extract preparation the resulting strain was grown normally at 30°C in YEPD media (prepared in house) in a batch fermenter to an optical density OD_600_ ∼2.4 – 3.2.

### Method details

#### Cloning and protein expression

*S. cerevisiae* Prp18 and Slu7, which form a heterodimer (James et al., 2002), were cloned into a pETDuet vector, modified in-house, and used for recombinant expression in *E. coli* BL21 (DE3) RIL cells. Cells were grown in LB media to OD_600_ ∼0.8 and expression was induced with 0.5 mM IPTG at 18°C for 16 hours. Purification was performed essentially as described (James et al., 2002). Cell pellets were lysed by sonication in buffer A250 (50mM Tris-HCl, pH 7.5, 250mM NaCl, 10mM Imidazole, 0.05% NP-40) and cell debris cleared by centrifugation at 15000 rpm in a Sorvall SS34 rotor. Supernatants were incubated with 1-2 mL Ni-NTA agarose at 4°C for 1 hour, beads washed with 50mL of buffer A250, and bound proteins eluted stepwise with 50mM, 100mM, and 200mM Imidazole in Buffer A250. Peak fractions containing the heterodimer, eluting at 100 mM Imidazole, were pooled and further purified by gel filtration chromatography in buffer A250. The purified heterodimer was dialysed against buffer D (20mM HEPES-KOH pH 7.9, 0.2mM EDTA, 1mM DTT, 20% glycerol, 100mM KCl) and stored at −80°C.

*S. cerevisiae* Prp16 K379A was cloned into a pRS424 vector and expressed in *S. cerevisiae* BCY123 cells, essentially as described (Galej et al., 2013). BCY123 cells were co-transfected with expression vectors pRS426 and pRS424, the latter coding for the recombinant protein. Positive transformants were grown in 12L YM4 selective media supplemented with 1% raffinose at 30°C to OD_600_ ∼0.9. Protein expression was induced with 2% galactose at 30°C for 18 hours. Cells were harvested, washed once with 1L deionised water, resuspended in 1 volume 2 x lysis buffer (100mM Tris-Cl pH 8.0, 1M NaCl, 2mM imidazole, 20mM β-mercaptoethanol (β-ME), 0.2% IGEPAL CA-630, 4mM CaCl_2_, 2mM MgCl_2_, EDTA-free protease inhibitor cocktail (Roche)), and frozen as droplets in liquid nitrogen. Frozen pellets were ground in a 6870 Freezer/Mill (SPEX SamplePrep) and ground powder was thawed at room temperature. Cell debris were removed by centrifugation at 15000 rpm in a Sorval SS34 rotor for 20 minutes. The lysate was further clarified by centrifugation at 40000 rpm for 45 minutes in a Beckman Ti 45 Rotor. The pH of the extract was adjusted to pH 7.5 by addition of 1M Tris base. The extract was incubated with 5 mL Calmodulin-Sepharose beads (home-made) for 12-16 hours at 4°C. Beads were washed 3 times with 25mL CAL500 buffer (20mM Tris-Cl pH 8.0, 500mM NaCl, 2mM CaCl_2_, 1mM MgCl_2_, 10mM β-ME) and the proteins eluted with CAL500 elution buffer (20mM Tris-Cl pH 8.0, 500mM NaCl, 2mM EGTA, 1mM MgCl_2_, 10mM β-ME). Protein-containing fractions were pooled and dialyzed against buffer Ni500 (20mM Tris-Cl pH 8.5, 500mM NaCl, 5mM imidazole, 10mM β-ME) overnight at 4°C. The dialysed protein was then incubated with 1mL Ni-NTA agarose beads at 4°C for 1 hour, and beads were washed three times with 10mL Ni500 wash buffer with 25mM imidazole. The protein was eluted with Ni500 elution buffer with 250mM imidazole. Peak fractions, containing pure Prp16 were pooled, dialyzed against buffer D200 (20mM HEPES-KOH pH 7.9, 200mM KCl, 0.2mM EDTA, 1mM DTT, 20% glycerol), and stored at −80°C.

#### C^prp16-302^ purification and biochemistry

Spliceosomes were assembled on a modified *UBC4* pre-mRNA with 25 nucleotide exons (Fica et al., 2013), labeled at the 3′ end with Fluorescein (Cy2), in extracts from *prp16-302* / *CWC25*-TAPS by incubation at 19°C for 30 minutes. Following splicing, reactions were incubated at 19°C for another 15 minutes in the presence of 0.2μM of a DNA oligo directed against the 5′-splice site, to degrade unassembled pre-mRNA, and 2mM glucose to deplete ATP and minimize Prp16 activity. Reactions were centrifuged through a 40% glycerol cushion in buffer K-75 (20mM HEPES-KOH pH 7.9, 75mM KCl, 0.25mM EDTA, 0.05% NP-40) and assembled complexes were affinity purified from the cushions using lgG Sepharose 6 Fast Flow (GE Healthcare) to capture the Protein A tag. Following extensive washing with buffer K-75, complexes were eluted from the beads by incubation with 25 ug/ mL TEV protease (expressed in-house) by incubation at room temperature (22-23°C) for one hour in the presence of 1mM DTT, and the protease was removed by subsequent concentration through a 50 kDa MWCO Amicon concentrator. Spliceosomes were further purified via the Strep II tag on Cwc25 using Streptactin affinity resin (GE) in buffer K-75 (20mM HEPES-KOH pH 7.9, 75mM KCl, 0.25mM EDTA, 5% glycerol, 0.05% NP-40) and eluted with 5mM desthiobiotin. Eluted complexes were concentrated in a 100 kDa MWCO Amicon concentrator to ∼5-10nM, buffer exchanged into buffer K-75 without glycerol (20mM HEPES-KOH pH 7.9, 75mM KCl, 0.25mM EDTA, 0.0025% NP-40), and used for EM data collection or biochemistry (Figure S1). This sample was used for EM dataset 4.

For biochemical assays, purified C complexes were chased at 26°C for 60 minutes in reactions containing 10% concentrated spliceosomes (∼100 fmoles, 2nM, in K-75 without glycerol), 3% PEG_8000_, 60 mM potassium phosphate buffer (pH 7.0), 2mM ATP (or ATP- γS), and 4mM MgCl_2_. Specific reactions were also supplemented with 250nM Slu7/ Prp18 heterodimer. RNAs were extracted and analyzed on a 14% denaturing polyacrylamide gel.

For EM data collection following biochemical chase, purified C complexes were incubated at 26°C with 2mM ATP and 2.5mM MgCl_2_ for 15 minutes prior to grid preparation. This sample was used for EM dataset 5.

For both un-chased and chased complexes, the purified sample was applied to Cu 300 R1.2/1.3 holey carbon grids (Quantifoil) coated with a ∼6 nm homemade carbon film. Grids were glow discharged for 30 s before application of 3.5 μL sample, then incubated for 25 s and blotted for 2.5-3.5 s before vitrification by plunging into liquid ethane using an FEI Vitrobot MKIII operated at 100% humidity and 4°C.

#### Modulation of branching reversibility in C complex

Extracts were prepared from a strain carrying an HA tag on Cwc25 (*Cwc25-HA*), which was a gift from S.-C. Cheng (Chiu et al., 2009). Splicing reactions were carried out on a 4mL scale in the presence of Prp16 K379A (added at 18 ng/ μL) and using 4 nM *UBC4* pre-mRNA substrate, labeled at the 3′ end with Fluorescein (Cy2) or Cy5. Reactions were incubated at 20°C for 45 minutes, then diluted and centrifuged through a 40% glycerol cushion in buffer K-75 (20mM HEPES-KOH pH 7.9, 75mM KCl, 0.25mM EDTA, 0.05% NP-40). Cushions were recovered and incubated with 150 μL of anti-HA Agarose (Sigma) at 4°C overnight (∼18 hours). Beads were washed 3 times with buffer NET-2 (50mM Tris-HCl pH 7.4, 150mM NaCl, 0.05% NP-40) and one time with 10mM Tris-HCl pH 8.6 and resuspended in 10 mM Tris-HCl pH 8.6. For single incubation experiments, spliceosomes were incubated with 4mM MgCl_2_ and 150mM monovalent ion chlorides at 26°C with rotation for one hour. For double incubation experiments, spliceosomes were incubated with 4mM MgCl_2_ and 150mM LiCl at 26°C with rotation for one hour. Beads were spun at 3000 rpm for 2 minutes and washed once with 10mM Tris-HCl pH 8.6, then resuspended in 10mM Tris-HCl pH 8.6 and chased with 4mM MgCl_2_ and 150mM monovalent ion chlorides at 26°C with rotation for one hour. RNAs were extracted and analyzed on a 14% denaturing polyacrylamide gel.

#### P-complex purification

Dataset 6 described in this paper derives from our early attempts to purify P complex. mRNA release was stalled by a dominant negative Prp22 mutant (K512A) and spliceosomes were purified first by mRNA pulldown then by Slu7-TAPS pulldown. This strategy produces a mixture of C and C^∗^/P complex and predates our discovery that RNaseH cleavage could be used to significantly enrich P complex in the final sample (Wilkinson et al., 2017) (Figure S1). In detail, Slu7-TAPS splicing extract was prepared as described (Galej et al., 2016). Splicing extracts were treated on ice for 10 min with 0.04 mg/mL recombinantly-expressed Prp22 K512A. Spliceosomes were assembled in this extract on a UBC4 pre-mRNA with two MS2 hairpins pre-bound to MS2-MBP fusion protein as described (Zhou et al., 2002) by incubation at 23°C for 30 minutes. Following splicing, reactions were centrifuged through a 40% glycerol cushion in buffer K-75 (20 mM HEPES KOH pH 7.9, 75 mM KCl, 0.25 mM EDTA, 0.05% NP-40) and assembled complexes were affinity purified from the cushions using amylose resin (New England Biolabs). Following washing with buffer K-75, complexes were eluted from the beads with 12 mM maltose. Spliceosomes were further purified via the Step II tag on Slu7 using Streptactin affinity resin (GE) in buffer K-100 (20 mM HEPES KOH pH 7.9, 100 mM KCl, 0.25 mM EDTA, 5% glycerol, 0.01% NP-40) and eluted with 5 mM desthiobiotin. Eluted complexes were concentrated in a 100 kDa MWCO Amicon concentrator and dialysed into buffer K-75 without glycerol (20 mM HEPES KOH pH 7.9, 75 mM KCl, 0.25 mM EDTA). This sample was used for EM dataset 6: the purified sample was applied to Cu 300 R1.2/1.3 holey carbon grids (Quantifoil) coated with a ∼7 nm homemade carbon film. Grids were glow discharged for 30 s before application of 3 μL sample, then incubated for 30 s and blotted for 2.5 s before vitrification by plunging into liquid ethane using an FEI Vitrobot MKIII operated at 100% humidity and 4°C

#### Cryo-EM data acquisition

Eight datasets and a total of 24,115 movies were collected manually or using EPU on various Titan Krios microscopes (Thermo Fisher) all equipped with energy filters (slit width of 20 eV) and K2 detectors operated in counting or super-resolution mode (Figure S2). Dataset 1 (2,213 movies) is described (Galej et al., 2016) and was collected manually on LMB Krios 1 in super-resolution mode with a real pixel size of 1.427 Å/pix, a defocus range of −0.5 to −4 μm, with an exposure time of 16 s fractionated into 20 frames and a total dose per micrograph of 40 e^-^/Å^2^. Datasets 2 and 3 were described previously (Fica et al., 2017). Dataset 2 (1,571 movies) was collected manually on LMB Krios 1 in super-resolution mode with a real pixel size of 1.427 Å/pix, a defocus range of −0.5 to −4.5 μm, with an exposure time of 16 s fractionated into 20 frames and a total dose per micrograph of 40 e^-^/Å^2^.

Dataset 3 (2,025 movies) was collected using EPU on Diamond Light Source (DLS) Krios 1 in counting mode with a pixel size of 1.031 Å/pix, a defocus range of −0.5 to −3.5 μm, with an exposure time of 14 s fractionated into 28 frames and a total dose per micrograph of 42 e^-^/Å^2^.

Dataset 4 (2,944 movies) was collected using EPU on Diamond Light Source (DLS) Krios 2 in counting mode with a pixel size of 1.023 Å/pix, a defocus range of −0.5 to −3.5 μm, with an exposure time of 14 s fractionated into 28 frames and a total dose per micrograph of 44 e^-^/Å^2^.

Dataset 5 (2,523 movies) was collected using EPU on LMB Krios 1 in counting mode with a pixel size of 1.12 Å/pix, a defocus range of −0.5 to −3 μm, with an exposure time of 14 s fractionated into 28 frames and a total dose per micrograph of 56 e^-^/Å^2^

Dataset 6 (4,369 movies) was collected using EPU on a Krios at SciLifeLab (Stockholm, Sweden) in counting mode with a pixel size of 1.028 Å/pix, a defocus range of −0.5 to −3.5 μm, with an exposure time of 8 s fractionated into 20 frames and a total dose per micrograph of 39.7 e^-^/Å^2^.

Dataset 7 (2,384 movies) is described in Wilkinson et al. (2017) and was collected using EPU on LMB Krios 1 in counting mode with a pixel size of 1.12 Å/pix, a defocus range of −0.2 to −3 μm, with an exposure time of 12 s fractionated into 20 frames and a total dose per micrograph of 47 e^-^/Å^2^.

Dataset 8 (1,614 movies) is described in Wilkinson et al. (2019) and was collected using EPU on LMB Krios 1 in counting mode with a pixel size of 0.88 Å/pix, a defocus range of −0.5 to −3.5 μm, with an exposure time of 7 s fractionated into 35 frames and a total dose per micrograph of 45.2 e^-^/Å^2^.

Dataset 9 was of a P complex sample prepared identically to Wilkinson et al. (2017) except that the final sample buffer contained 1 mM MgCl_2_ instead of 0.25 mM EDTA. 4830 micrographs were collected using EPU on a Diamond Light Source Krios in counting mode with a pixel size of 1.03 Å/pix, a defocus range of −0.5 to −3 μm, with an exposure time of 12 s fractionated into 40 frames and a total dose per micrograph of 49.2 e^-^/Å^2^.

#### Initial cryo-EM data processing

All datasets were initially processed separately (Figure S2). Dataset 1 was processed as described previously (Galej et al., 2016) yielding a 3.8 Å reconstruction of C complex (EMD-4055). Re-refining these particles with a mask on the spliceosome core improved the resolution to 3.61 Å. Datasets 2 and 3 were processed as described previously (Fica et al., 2017), yielding both a reconstruction of C^∗^ complex at 3.8 Å resolution (EMDB-3539) and reconstructions of C complex at 4.62 Å resolution (dataset 2) and 3.61 Å resolution (dataset 3).

Datasets 4 and 5 were processed similarly: particles were picked using RELION auto-pick using representative 2D class averages of C complex as templates. After 3D classification to select good quality particles, particles were polished using the method implemented in RELION 1.3 (Scheres, 2014), i.e., not the Bayesian polishing routine of RELION 3.0. Masked refinement then yielded C complex reconstructions at 3.34 Å resolution (dataset 4) and 3.37 Å resolution (dataset 5).

Dataset 6 was processed similarly to datasets 4 and 5 except that due to splicing being stalled prior to mRNA release, approximately half the particles were in the exon-ligation conformation (C^∗^/P) and half were in the branching conformation (C). 3D classification was used to resolve these different populations, and each was subjected to particle polishing in RELION 2.0, producing a 3.58 Å resolution reconstruction of C^∗^/P complexes, and 3.42 Å resolution reconstruction of C complex.

Dataset 7 (Wilkinson et al., 2017) was reprocessed in RELION 3.1. After motion-correction with dose-weighting, particles were picked with crYOLO (Wagner et al., 2019) using a model trained on the dataset. After 3D classification to select good P complex particles, particles were subject to CTF refinement and Bayesian polishing, producing a P complex reconstruction at 3.50 Å resolution.

Dataset 8 was processed as described in Wilkinson et al. (2019), yielding a 3.56 Å reconstruction of P complex.

Dataset 9 processed in RELION 3.1. After motion-correction with dose-weighting, particles were picked with crYOLO (Wagner et al., 2019) using a general model. After 3D classification to select good P complex particles, particles were subject to CTF refinement and Bayesian polishing, producing a P complex reconstruction at 3.13 Å resolution.

#### C-complex data processing

##### Initial refinement

C complex data were merged using RELION 3.1, using separate optics groups for datasets 1, 2, 3, 4, 5, and 6 (Figure S2). Dataset 1 was further split into 3 optics groups as this dataset was collected over three different microscope sessions. The individual reconstructions were compared with UCSF Chimera to determine the relative pixel sizes of each dataset, and defocus values were scaled by the relative difference in pixel sizes (Wilkinson et al., 2019). Unscaled particles were then refined together with a mask on the ordered core, with RELION 3.1 internally scaling the particles to match a 1.12 Å/pix reference in a 400 pixel box. Two rounds of CTF refinement (refining per-particle defocus, per-micrograph astigmatism and B-factor, and per-optics group anisotropic magnification, beam tilt, trefoil, and 4^th^ order aberrations) produced a reconstruction at 2.80 Å resolution (Figure S4). The reconstruction had a good angular distribution that was improved by merging the 6 datasets (Figure S4). Density modification in Phenix (Terwilliger et al., 2020) further improved the map resolution to 2.69 Å.

##### Focused refinements

Although the core of the C-complex spliceosome is well ordered, many of the peripheral domains are flexible and had weak EM density in the overall refinement map, and correspondingly the local resolution quickly decayed toward the edge of the spliceosome (Figure S4). For each domain, strategies including signal subtraction, classification without alignment, and focused refinement with and without the reconstruction algorithm SIDESPLITTER (Ramlaul et al., 2020) were systematically investigated. All signal subtractions started from a 3 Å resolution map of C complex where the refinement mask encompassed the entire complex. All focused refinements used solvent-flattened FSCs to calculate the resolution during refinement. The following strategies produced the best maps for each domain, and are summarized in Figure S3 and Table S1.

Density for the U2 snRNP core domain was improved first by signal subtraction with a soft mask (8 pixel hard edge, 16 pixel soft edge) encompassing the U2 snRNP core, Prp8 RNaseH domain and Syf1 N terminus. Signal-subtracted particles were shifted to the mask center-of-mass and cropped to a 200 pixel box. 3D classification without alignment and T = 4 into 4 classes required 60 iterations for convergence and showed that 26% of particles (108,540) did not have strong U2 snRNP density. These particles were removed, and the resultant STAR file was reverted to the original, un-subtracted particles. These were focus-refined using the same mask used for signal subtraction, using the overall map low-pass filtered to 7 Å as a reference and performing local angular searches starting at 0.9 degree sampling. This produced a reconstruction at 4.07 Å resolution. Refinement was then continued using a tighter mask that only encompassed the U2 snRNP core (3 pixel hard edge, 16 pixel soft edge), using a 5 Å resolution reference and performing local angular searches at 0.5 degree sampling. This produced a map for the U2 snRNP at 3.58 Å resolution. Density modification in Phenix (Terwilliger et al., 2020) further improved the map resolution to 3.26 Å.

The NTC TPR domains (Syf1 and Clf1 and associated proteins) was improved first by signal subtraction with a mask (1 pixel hard edge, 8 pixel soft edge) encompassing the entirety of Clf1, Syf1 and the associated proteins. Signal-subtracted particles were shifted to the mask center-of-mass and cropped to a 300 pixel box. 3D classification without alignment and T = 4 into 4 classes required 25 iterations for convergence and showed that 43% of particles (172,895) did not have strong density for the peripheral helical arches. These particles were removed, and the remaining particles were focus-refined using a soft mask (8 pixel hard edge, 16 pixel soft edge) that only encompassed the flexible peripheral helical arches, e.g., excluding the well resolved N terminus of Clf1. The best 3D class, low-pass filtered to 8 Å, was used as a reference, local angular searches started at 0.9 degree sampling, and the external reconstruction program SIDESPLITTER was used to reduce overfitting. This produced a reconstruction at 3.82 Å resolution. Refinement was then continued for 3 more iterations with the same mask, using a 3.8 Å resolution reference and performing local angular searches with 0.9 degree sampling. This produced an improved reconstruction with smoother densities, although the resolution stayed constant at 3.82 Å. Density modification in Phenix (Terwilliger et al., 2020) then further improved the map resolution to 3.50 Å.

Despite numerous attempts, the helicase module of C complex, consisting of Brr2, Prp16, and the Prp8 Jab1/MPN domain, could not be improved to near-atomic resolution. This module is larger than the U2 snRNP core which was successfully refined to near-atomic resolution. Therefore, this module may have internal flexibility, e.g., between the RecA domains of Brr2 and Prp16, that makes particles difficult to align. Nevertheless, densities were made more interpretable as follows. First signal subtraction was performed using a mask (1 pixel hard edge, 8 pixel soft edge) loosely encompassing Brr2, Prp16, and Prp8 Jab1/MPN. Signal-subtracted particles were shifted to the mask center-of-mass and cropped to a 300 pixel box. 3D classification without alignment and T = 4 into 4 classes required 60 iterations for convergence and showed that only 26% of particles (103,461) had strong density for the helicase module. These were focus refined with a very soft mask (16 pixel hard edge, 12 pixel soft edge), using 0.9 degree local angular sampling, and the best class low-pass filtered to 20 Å as a reference. Postprocessing with a tighter mask (1 pixel hard edge, 16 pixel soft edge) gave a reconstruction of the helicase module at 8 Å resolution, which was used for interpreting Prp16. Classification without alignment suggested that Brr2 and Prp8 Jab1/MPN was flexible relative to Prp16. Therefore, a further focused refinement was performed using a soft mask (8 pixel hard edge, 16 pixel soft edge) just around Brr2 and Prp8 Jab1/MPN, with a 20 Å resolution reference, 0.9 degree local angular sampling, and an initial offset search range of 3 pixels, producing a map at 7.15 Å resolution. Similar attempts to perform focused refinement on just Prp16 were not successful.

In the overall C-complex map, the WD40 domain of Prp17 was weakly defined and did not allow unambiguous docking of the model from P complex, with all 14 possible orientations of the 7-bladed beta propeller being consistent with the density. However, this domain was too small for focused refinement. Instead, to improve the local resolution we performed multiple rounds of classification without alignment and with high T values to select for high-resolution subsets of particles with Prp17 in similar positions. First, signal subtraction was performed using a loose spherical mask (12 pixel hard edge, 8 pixel soft edge) around the WD40 domain and some neighboring proteins. Signal-subtracted particles were shifted to the mask center-of-mass and cropped to a 200 pixel box. 3D classification without alignment and T = 50 into 4 classes required 60 iterations for convergence. The single class with the most well-defined density (24%, 96,800 particles) was selected and 3D classification without alignment was repeated using a tighter mask (1 pixel hard edge, 8 pixel soft edge), T = 50, and 4 classes. Again, the class with best defined density (58%, 59,152 particles) was selected. The selected particles were then reverted to the un-subtracted particles, and half maps were reconstructed from the original Euler angles using relion_reconstruct. Postprocessing with a mask encompassing the entire C complex produced a map at overall 3.67 Å resolution. The local resolution around the Prp17 WD40 domain was still limited, but now allowed unambiguous docking of the model from P complex, with one of the 14 possible orientations giving clearly higher correlations and atom inclusion scores when docking in UCSF Chimera.

##### Focused classification of step II factors

To obtain robust estimates of the occupancy of the step II factors Prp18 and Slu7 on the C complex spliceosome, we used multiple parallel 3D classifications and merged selected classes (Figure S3). To identify particles containing the Slu7 zinc knuckle domain, signal subtraction was used with a small spherical mask (8 pixel hard edge, 8 pixel soft edge) centered on Slu7. Signal-subtracted particles were shifted to the mask center-of-mass and cropped to a 200 pixel box. Two parallel 3D classifications without alignment were performed, both into 4 classes with 60 iterations and using a tighter spherical mask (1 pixel hard edge, 8 pixel soft edge). One classification used T = 100, the other used T = 1000. The T = 100 classification showed 29% of particles contained the zinc knuckle domain, and the T = 1000 classification showed that 18% of particles contained the same domain. After merging these particles and removing duplicates, 33% (130,588) of the original particles were captured, and further classification without alignment did not result in further segregation into different classes: using T values ranging from 100 to 2000, 85 – 95% of particles would converge on a single class with strong zinc knuckle density. Therefore, these 33% were taken as the final “Slu7 zinc knuckle” class.

To identify particles containing Prp18 and the associated EIE region of Slu7, 3D classification was performed both with varying T values and with and without signal subtraction. Signal-subtracted particles were generated using a soft mask centered on Prp18 (4 pixel hard edge, 12 pixel soft edge), and were shifted to the mask center-of-mass and cropped to a 200 pixel box. Two parallel 3D classifications without alignment were performed on the signal-subtracted particles, both into 4 classes and using the same mask as for signal subtraction. One classification used T = 4 and 60 iterations, the other used T = 10 and 120 iterations. The T = 4 classification showed 20% (82,492) of particles with strong density for Prp18, and the T = 10 classification showed that 20% (81,431) of particles contained the same domain. These subsets were merged and duplicate particles were removed, yielding 23% (91,424) of the total particles.

Two parallel 3D classifications without alignment were also performed on the non-signal-subtracted particles, both into 4 classes with 60 iterations and using the same mask as for the signal-subtracted particles. One classification used T = 4 and showed 20% (82,398) of particles with strong Prp18 density, the other classification used T = 20 and showed 21% (82,872) of particles with strong Prp18 density and 21% (83,389) with some weak Prp18 density. These subsets were merged with the 91,424 un-subtracted particles from the signal-subtracted classifications, and duplicate particles were merged, yielding 186,323 particles. These were subjected to a final 3D classification without alignment into 4 classes, using a looser mask (8 pixel hard edge, 8 pixel soft edge), 60 iterations, and T = 20. Two of the resulting classes had strong Prp18 density and were selected as the final “strong Prp18” class, containing 120,141 particles, or 30% of the original particles.

Contingency tables for each dataset (Figure S7) were determined from the above classifications, i.e., the datasets were not individually classified.

##### Merging C and P complex data to improve Prp19, Cwc22, and U5 Sm reconstructions

The Prp19 module, U5 Sm domain, and the N-terminal domain of Cwc22, are each very flexible but are in similar positions in C and P complexes as they are unaffected by the conformational change between step I and step II of splicing. We therefore reasoned that the best resolutions for these regions could be obtained by merging C and P complex data to give high initial particle numbers for focused classification.

First, all P complex data were first merged using RELION 3.1, using separate optics groups for datasets 6, 7, 8, and 9 (Figure S2). The individual reconstructions were compared with UCSF Chimera to determine the relative pixel sizes of each dataset, and defocus values were scaled by the relative difference in pixel sizes (Wilkinson et al., 2019). Unscaled particles were then refined together, with RELION 3.1 internally scaling the particles to match a 1.12 Å/pix reference in a 400 pixel box. Two rounds of CTF refinement (refining per-particle defocus, per-micrograph astigmatism, and per-optics group anisotropic magnification, beam tilt, trefoil, and 4^th^ order aberrations) followed by a final masked refinement produced a reconstruction at 2.95 Å resolution from 320,770 particles. This map had unclear density for the docked 3′ splice site, so we still consider our previous reconstruction at 3.30 Å with strong 3′ splice site density as the best “reference” P complex structure (Wilkinson et al., 2019).

Next, we merged all our C complex particles with these P complex particles described above, giving a total of 724,244 particles that when refined produced a map that largely resembled C complex (presumably due to the majority of the particles – 56% – being in the step I conformation) although with weaker density for the mobile U2 snRNP, the branch helix, and the step I factors (Figure S3). For the extremely mobile Prp19 domain of the NTC, no focused refinements were successful. We therefore performed signal subtraction with a loose mask that would encompass most possible orientations of this domain, constructed by summing all classes from a preliminary classification without alignment, then adding a 2 pixel hard edge and 8 pixel soft edge. Signal-subtracted particles were shifted to the mask center-of-mass and cropped to a 300 pixel box. 3D classification without alignment and T = 70 into 12 classes showed there were indeed many possible orientations of this domain (Figure S3). The most stable class, containing 7% of the original particles (49,514) was selected. Half maps were reconstructed for the signal-subtracted particles using relion_reconstruct and the original Euler angles, allowing postprocessing with a soft mask (hard edge 8 pixels, soft edge 16 pixels) encompassing just the stable orientation, which produced a map at nominally 7.30 Å resolution. This resolution is probably overestimated but the map nonetheless shows some secondary structure features consistent with the expected helical bundle (Figure S5). We also reverted to the original C-complex particles (35,716) and calculated half-maps with relion_reconstruct to show how this stable orientation relates to the body of the spliceosome. After postprocessing with a mask around the entire C-complex, this produced a reconstruction at 3.97 Å resolution (local resolution around Prp19 ∼8 Å) showing how the N terminus of Prp46 probably projects into the Prp19 module.

A similar approach was applied for the U5 Sm domain (Figure S3), as this domain was too small for focused refinements. Signal subtraction was performed on the combined C/C^∗^/P particles using a soft mask encompassing the U5 Sm ring and the base of U5 snRNA (hard edge 4 pixels, soft edge 8 pixels), and re-centering particles on the mask center-of-mass and cropping to a 200 pixel box. Two rounds of 3D classification without alignment were used to select the particles with the most stably-bound, highest resolution U5 Sm domain. The first round with T = 20 eliminated 54% of particles which showed no density for the U5 Sm domain, and the second round with T = 100 selected 23% of particles (65,561 particles, 9% of original particles) which were used to calculate half-maps with relion_reconstruct using the original Euler angles. After postprocessing with a mask around the entire spliceosome, this produced a reconstruction at 3.13 Å resolution with much stronger density for the U5 Sm ring than the original map. The map was further improved by density modification in Phenix (Terwilliger et al., 2020) to 2.96 Å resolution, which smoothened some of the discontinuous densities in the peripheral Sm chains.

Finally, a similar approach was utilized for the N-terminal domain of Cwc22 (Figure S3). Signal subtraction was performed on the combined C/C^∗^/P particles using a soft mask encompassing the Cwc22 NTD (hard edge 4 pixels, soft edge 8 pixels), and re-centering particles on the mask center-of-mass and cropping to a 200 pixel box. Two rounds of 3D classification without alignment were used to select the most stable, high-resolution position of Cwc22 NTD. The first round with T = 100 eliminated 72% of particles which showed no density for Cwc22 NTD, and the second round with T = 1000 selected 46% of particles (73,802 particles, 10% of original particles) which were used to calculate half-maps with relion_reconstruct using the original Euler angles. After postprocessing with a mask around the entire spliceosome, this produced a reconstruction at 3.16 Å resolution. The map was further improved by density modification in Phenix (Terwilliger et al., 2020) to 2.97 Å resolution.

#### C-complex model building

To avoid being biased by our previous model of C complex (Galej et al., 2016) which was built into lower resolution density (3.8 Å average resolution), the entire ordered core of C complex was rebuilt entirely *de novo* in Coot (Casañal et al., 2020), based only on the 2.8 Å cryo-EM density. This included the proteins Prp8 (except the RNase H and Jab1/MPN domains), Snu114, Prp45, Prp46, Ecm2, Cwc2, Cwc15, Bud31, Cef1, Clf1, Syf1, Syf2, Cwc21, Cwc22, Yju2, Cwc25, Isy1, and the U2, U5, and U6 snRNAs, the intron, and the 5′ exon. To improve model geometry, we compared the scale of the map to crystal structures of Prp8 (PDB 4I43; Galej et al., 2013) and the NMR structure of Bud31 (PDB 2MY1) (van Roon et al., 2015), which showed that the true pixel size of the map should be 1.145 Å/pix. The model coordinates were all scaled by 1.145/1.12 = 1.02232 before further model building.

ISOLDE (Croll, 2018) was then used to diagnose and fix errors and improve Ramachandran, CaBLAM, and rotamer outliers. The resultant core model largely resembled our earlier model, but with more accurate backbone geometry and rotamer assignment (Table S1), and some fixing of register errors including in Cef1 helix residues 230-249 and the Cwc15 N terminus. Notable areas of improvement include the C-terminal domain of Ecm2, the interface of U2 snRNA stem IIb with Cwc2, domain IV of Snu114, assignment of the C-terminal helices of Yju2, and assignment of the very N terminus of Prp45 and an N-terminal helix of Prp46 projecting toward Prp19.

For building into the focused-refined maps, these maps were first all aligned and resampled in Chimera to their average positions, as determined from their most populated 3D classes. phenix.combine_focused_maps was then used to make a composite map from the resampled, density-modified maps for the core (2.69 Å), U2 snRNP (3.26 Å), NTC (3.50 Å), U5 Sm (2.96 Å) and Cwc22 NTD (2.97 Å), and models were built into this composite map.

The helical arches of Clf1 and Syf1 were built *de novo* into the NTC part of the composite map using Coot. The map was of sufficient quality to allow unambiguous building of the N-terminal half of Clf1 and the middle region of Syf1 (Figure S5). However, the local resolution still deteriorated toward the periphery of the focus-refined map (Figure S4), so we used secondary structure predictions generated by the GeneSilico MetaServer (Kurowski and Bujnicki, 2003) and cross-linking data from yeast B^act^ complex (Rauhut et al., 2016) to support our building. Multiple sequence alignment was also useful, revealing a yeast-specific insertion in Syf1 that we predicted formed a disordered loop, since the cryo-EM density of human Syf1 in human P complex (Fica et al., 2019) largely resembled the cryo-EM density for yeast Syf1. The completed Syf1 model left several helical densities unassigned, with no topological means of filling them with Syf1 sequence. Based on further secondary structure predictions, crosslinking in yeast B^act^ complex, and in some cases clear side-chain density, we assigned these densities to Ntc20, Isy1, and Syf2. This left two remaining helices. One was tentatively assigned to Cef1 but was built with UNK residues, and the other is left unassigned (chain X, UNK residues). The model was then improved in ISOLDE (Croll, 2018).

The Sm domain of the U2 snRNP was built by docking in the structure of the yeast U1 snRNP Sm domain from pre-B complex (Bai et al., 2018) and manually adjusting all side chains and loops to fit the density using Coot. Homology models for Lea1 and Msl1 were generated with I-TASSER (Yang et al., 2015) based on the structure of human U2A′/U2B″ in complex with U2 snRNA (PDB 1A9N; Price et al., 1998) and were docked into the density and manually fixed. Density connecting the U2 snRNP to Syf1 could not be accounted for by Syf1 helices, and was assigned to Isy1 and the C terminus of Lea1 based on side-chain density and crosslinking data from yeast B^act^ complex. Double-stranded RNA density corresponding to stems IV, V and the 950 nt yeast-specific insertion in U2 snRNA, was modeled by generating idealized structures in RNAComposer (Popenda et al., 2012) and adjusting to the density in Coot. The model was then improved using ISOLDE, with adaptive distance restraints used to maintain pairing in U2 stems IV and V.

The Sm domain of U5 snRNP was built by copying our new model for the U2 snRNP Sm domain and docking into the composite map. The associated region of U5 snRNA was docked from yeast B complex (Plaschka et al., 2017). The fit for both was improved used using ISOLDE, with adaptive distance restraints on the Sm ring using U2 snRNP as a template, and strong adaptive distance restraints (kappa value of 50) on U5 snRNA.

The Cwc22 N-terminal domain was built by docking an I-TASSER model based on the crystal structure of human Cwc22 complexed with eIF4AIII (PDB 4C9B) (Buchwald et al., 2013) into the composite map. Extraneous loops were truncated and the fit was improved with ISOLDE.

Prp17, the Prp8 RNase H domain, Prp18, and Slu7 all have stronger densities in P complex than in C complex. These were therefore rebuilt using ISOLDE into our newer 3.3 Å map (EMD-10140) (Wilkinson et al., 2019), scaled up to 1.145 Å/pix from 1.12 Å/pix, starting from our original deposited 3.7 Å P-complex model (PDB 6EXN) (Wilkinson et al., 2017). Prp17, Slu7 zinc-knuckle, and Prp8 RNase H + Prp18 + Slu7 EIE region were each docked into their respective focus-classified C-complex maps, and ISOLDE was used to fix any clashes and improve the fit to density.

For the helicase domain, a composite map was prepared with phenix.combine_focused_maps from the overall helicase focused refinement, which had best density for Prp16, and the Brr2 focused refinement, which had better density for Brr2 and Prp8 Jab1. The crystal structure of Brr2 associated with the Jab1/MPN domain of Prp8 (PDB 4BGD) (Nguyen et al., 2013) was docked into this composite map after minor rebuilding into the original structure factors with ISOLDE and refinement with phenix.refine to improve starting geometry. A homology model of Prp16 was generated with I-TASSER, with the top two templates being Prp43 (PDB 3KX2) (He et al., 2010) and MLE helicase (PDB 5AOR), and was docked into the composite map. ISOLDE with adaptive distance restraints was used to morph these starting models into the density. Very strong restraints (kappa of 20) were used for Brr2/Jab1 since the starting model was known to be of good quality. For Prp16, the individual domains (RecA1, RecA2, and CTD) were individually restrained with kappa of 10 to allow movement between them. The resulting Brr2 model was largely unchanged, but Prp16 adopted an open conformation that strongly resembled the crystal structure of *Chaetomium thermophilum* Prp22 in complex with RNA (PDB 6I3P) (Hamann et al., 2019). Indeed, equivalent density to the Prp22-bound RNA was observed next to the RecA2 domain, which based on distance to the modeled intron in the spliceosome core was assigned as residues 87 – 92 of the UBC4 intron (+17 to +22 relative to the branch point, −9 to −4 relative to the 3′ splice site). Prp16 and this region of the intron were then further refined in ISOLDE using strong adaptive distance restraints to the *Ct*Prp22-RNA crystal structure. Finally, the density did not allow correction of Ramachandran and CaBLAM outliers which were inherited from the source structures, but all rotamer outliers were manually corrected when possible.

The Prp19 module, consisting of the Prp19 tetramer, Snt309, and Cef1 C terminus, was modeled by first rebuilding our earlier model of the human ortholog in human P complex (Fica et al., 2019) using ISOLDE. An extra helix was built as chain X, residues UNK, but is likely the N terminus of Prp46. Multiple sequence alignments were then used to mutate and truncate this model to fit the yeast sequences using phenix.sculptor. The U-box domains of Prp19 were modeled using crystal structures of the yeast protein (PDB 2BAY) (Vander Kooi et al., 2006). This model was then docked into the 7.3 Å focused classification maps of the Prp19 module. This map was good enough to allow improvement of the fit using ISOLDE, using adaptive distance restraints to the starting positions, except for the U-box domains which were strongly restrained (kappa = 50) to the crystal structure positions. This fitting showed a pronounced bend in one of the Prp19 dimeric coiled-coils compared to the human structure. After fitting, a large portion of unassigned density remained between the Prp19 module and Syf1. Although low resolution, this density neatly accommodates the crystal structure of the Prp19 WD40 domain (PDB 3LRV) (Vander Kooi, 2010), which was docked in after rebuilding in ISOLDE and refinement into the original structure factors using phenix.refine. The Prp19 tetramer should have four such domains, and previous cryo-EM structures have shown these in a wide variety of positions, generally without strong support from density. The current assignment of the WD40 domain should also be viewed as tentative, although more well-supported than previous assignments.

#### C-complex model refinement

Coordinates for the core of C complex, U2 snRNP, NTC, U5 Sm, and Cwc22N were refined simultaneously in real space using phenix.real_space_refine in PHENIX (Afonine et al., 2018) into a composite map of the 5 respective density-modified focused maps. Base-pairing, base-stacking, and metal-coordination restraints were not imposed for the core, where the density was good enough to obtain better geometry and fit to density in the absence of external restraints. For refinement of the lariat-intron 2′-5′ linkage a custom set of restraints was adapted from the geometry of the 3′-5′ phosphodiester RNA backbone. Base-pairing and base-stacking restraints were however imposed for U5 snRNA near the U5 Sm site and U2 snRNA stems IIb/c, IV and V. We found that the default real_space_refine settings consistently worsened the excellent starting geometry of our models from ISOLDE. Systematic investigation found that performing one macro-cycle of global minimization and ADP refinement, skipping local grid searches, with a nonbonded weight of 2000 and overall weight of 0.5, gave the best model quality statistics (Table S1). The Prp19 module, helicase module, and Prp17 WD40 domain were not refined in PHENIX, but ISOLDE was used to resolve any clashes between these modules and the refined structure. The final C complex model comprises 45 protein chains, 3 snRNAs, and the intron and 5′ exon.

#### Data visualization

All structural figures were generated with UCSF ChimeraX (Goddard et al., 2018).

### Quantification and statistical analysis

The data in Figures 2 and 4 were quantified using an Amersham Typhoon imaging system and ImageQuant TL. The rolling ball algorithm was used for background subtraction. Bands for individual splicing species were first normalized to the total signal from all splicing species in each lane to obtain percentages, which were then used for calculating the efficiency of exon ligation and of pre-mRNA formation by reversal of branching from the lariat-intermediate. Quantifications in Figure 2F were obtained from four independent spliceosome immunoprecipitations using splicing extract obtained from one batch of yeast cells. Quantifications in Figures 4E and 4F were obtained from four independent spliceosome purifications using extracts from two independent biological replicates (independent extract preparations from two different batches of fermenter growth).

Statistical validation for the final model deposited in the PDB (PDB 7B9V) was performed using PHENIX (Tables 1 and S1) (Afonine et al., 2018).

## Supplemental information

Document S1. Figures S1–S7 and Table S1

Data S1. PyMol session for the composite C_i_-complex model, related to Figure 1

Document S2. Article plus supplemental information

## References

[bib1] Afonine P.V., Poon B.K., Read R.J., Sobolev O.V., Terwilliger T.C., Urzhumtsev A., Adams P.D. (2018). Real-space refinement in PHENIX for cryo-EM and crystallography. Acta Crystallogr. D Struct. Biol..

[bib2] Auffinger P., Grover N., Westhof E., Sigel A., Sigel H., Sigel R.K.O. (2011). 1:Metal Ion Binding to RNA. Structural and Catalytic Roles of Metal Ions in RNA.

[bib3] Bai R., Yan C., Wan R., Lei J., Shi Y. (2017). Structure of the Post-catalytic Spliceosome from Saccharomyces cerevisiae. Cell.

[bib4] Bai R., Wan R., Yan C., Lei J., Shi Y. (2018). Structures of the fully assembled *Saccharomyces cerevisiae* spliceosome before activation. Science.

[bib5] Bertram K., El Ayoubi L., Dybkov O., Agafonov D.E., Will C.L., Hartmuth K., Urlaub H., Kastner B., Stark H., Lührmann R. (2020). Structural Insights into the Roles of Metazoan-Specific Splicing Factors in the Human Step 1 Spliceosome. Mol. Cell.

[bib6] Brys A., Schwer B. (1996). Requirement for SLU7 in yeast pre-mRNA splicing is dictated by the distance between the branchpoint and the 3′ splice site. RNA.

[bib7] Buchwald G., Schüssler S., Basquin C., Le Hir H., Conti E. (2013). Crystal structure of the human eIF4AIII-CWC22 complex shows how a DEAD-box protein is inhibited by a MIF4G domain. Proc. Natl. Acad. Sci. USA.

[bib8] Burgess S.M., Guthrie C. (1993). A mechanism to enhance mRNA splicing fidelity: the RNA-dependent ATPase Prp16 governs usage of a discard pathway for aberrant lariat intermediates. Cell.

[bib9] Casañal A., Lohkamp B., Emsley P. (2020). Current developments in Coot for macromolecular model building of Electron Cryo-microscopy and Crystallographic Data. Protein Sci..

[bib10] Chan S.P., Cheng S.-C. (2005). The Prp19-associated complex is required for specifying interactions of U5 and U6 with pre-mRNA during spliceosome activation. J. Biol. Chem..

[bib11] Chan S.P., Kao D.-I., Tsai W.-Y., Cheng S.-C. (2003). The Prp19p-associated complex in spliceosome activation. Science.

[bib12] Chang K.-J., Chen H.-C., Cheng S.-C. (2009). Ntc90 is required for recruiting first step factor Yju2 but not for spliceosome activation. RNA.

[bib13] Chen C.H., Tsai W.Y., Chen H.R., Wang C.H., Cheng S.C. (2001). Identification and characterization of two novel components of the Prp19p-associated complex, Ntc30p and Ntc20p. J. Biol. Chem..

[bib14] Chiang T.-W., Cheng S.-C. (2013). A weak spliceosome-binding domain of Yju2 functions in the first step and bypasses Prp16 in the second step of splicing. Mol. Cell. Biol..

[bib15] Chiu Y.F., Liu Y.C., Chiang T.W., Yeh T.C., Tseng C.K., Wu N.Y., Cheng S.C. (2009). Cwc25 is a novel splicing factor required after Prp2 and Yju2 to facilitate the first catalytic reaction. Mol. Cell. Biol..

[bib16] Croll T.I. (2018). ISOLDE: a physically realistic environment for model building into low-resolution electron-density maps. Acta Crystallogr. D Struct. Biol..

[bib17] Eysmont K., Matylla-Kulińska K., Jaskulska A., Magnus M., Konarska M.M. (2019). Rearrangements within the U6 snRNA Core during the Transition between the Two Catalytic Steps of Splicing. Mol. Cell.

[bib18] Fabrizio P., Dannenberg J., Dube P., Kastner B., Stark H., Urlaub H., Lührmann R. (2009). The evolutionarily conserved core design of the catalytic activation step of the yeast spliceosome. Mol. Cell.

[bib19] Fica S.M., Nagai K. (2017). Cryo-electron microscopy snapshots of the spliceosome: structural insights into a dynamic ribonucleoprotein machine. Nat. Struct. Mol. Biol..

[bib20] Fica S.M., Tuttle N., Novak T., Li N.-S., Lu J., Koodathingal P., Dai Q., Staley J.P., Piccirilli J.A. (2013). RNA catalyses nuclear pre-mRNA splicing. Nature.

[bib21] Fica S.M., Mefford M.A., Piccirilli J.A., Staley J.P. (2014). Evidence for a group II intron-like catalytic triplex in the spliceosome. Nat. Struct. Mol. Biol..

[bib22] Fica S.M., Oubridge C., Galej W.P., Wilkinson M.E., Bai X.-C., Newman A.J., Nagai K. (2017). Structure of a spliceosome remodelled for exon ligation. Nature.

[bib23] Fica S.M., Oubridge C., Wilkinson M.E., Newman A.J., Nagai K. (2019). A human postcatalytic spliceosome structure reveals essential roles of metazoan factors for exon ligation. Science.

[bib24] Galej W.P., Oubridge C., Newman A.J., Nagai K. (2013). Crystal structure of Prp8 reveals active site cavity of the spliceosome. Nature.

[bib25] Galej W.P., Wilkinson M.E., Fica S.M., Oubridge C., Newman A.J., Nagai K. (2016). Cryo-EM structure of the spliceosome immediately after branching. Nature.

[bib26] Galej W.P., Toor N., Newman A.J., Nagai K. (2018). Molecular Mechanism and Evolution of Nuclear Pre-mRNA and Group II Intron Splicing: Insights from Cryo-Electron Microscopy Structures. Chem. Rev..

[bib27] Goddard T.D., Huang C.C., Meng E.C., Pettersen E.F., Couch G.S., Morris J.H., Ferrin T.E. (2018). UCSF ChimeraX: meeting modern challenges in visualization and analysis. Protein Sci..

[bib28] Haack D.B., Toor N. (2020). Retroelement origins of pre-mRNA splicing. Wiley Interdiscip. Rev. RNA.

[bib29] Hamann F., Enders M., Ficner R. (2019). Structural basis for RNA translocation by DEAH-box ATPases. Nucleic Acids Res..

[bib30] Hardy S.F., Grabowski P.J., Padgett R.A., Sharp P.A. (1984). Cofactor requirements of splicing of purified messenger RNA precursors. Nature.

[bib31] Haselbach D., Komarov I., Agafonov D.E., Hartmuth K., Graf B., Dybkov O., Urlaub H., Kastner B., Lührmann R., Stark H. (2018). Structure and Conformational Dynamics of the Human Spliceosomal B^act^ Complex. Cell.

[bib32] He Y., Andersen G.R., Nielsen K.H. (2010). Structural basis for the function of DEAH helicases. EMBO Rep..

[bib33] James S.-A., Turner W., Schwer B. (2002). How Slu7 and Prp18 cooperate in the second step of yeast pre-mRNA splicing. RNA.

[bib34] Jones M.H., Frank D.N., Guthrie C. (1995). Characterization and functional ordering of Slu7p and Prp17p during the second step of pre-mRNA splicing in yeast. Proc. Natl. Acad. Sci. USA.

[bib35] Kimanius D., Forsberg B.O., Scheres S.H., Lindahl E. (2016). Accelerated cryo-EM structure determination with parallelisation using GPUs in RELION-2. eLife.

[bib36] Krishnan R., Blanco M.R., Kahlscheuer M.L., Abelson J., Guthrie C., Walter N.G. (2013). Biased Brownian ratcheting leads to pre-mRNA remodeling and capture prior to first-step splicing. Nat. Struct. Mol. Biol..

[bib37] Kurowski M.A., Bujnicki J.M. (2003). GeneSilico protein structure prediction meta-server. Nucleic Acids Res..

[bib38] Lardelli R.M., Thompson J.X., Yates J.R., Stevens S.W. (2010). Release of SF3 from the intron branchpoint activates the first step of pre-mRNA splicing. RNA.

[bib39] Leontis N.B., Stombaugh J., Westhof E. (2002). The non-Watson-Crick base pairs and their associated isostericity matrices. Nucleic Acids Res..

[bib40] Lesser C.F., Guthrie C. (1993). Mutational analysis of pre-mRNA splicing in Saccharomyces cerevisiae using a sensitive new reporter gene, CUP1. Genetics.

[bib41] Lin R.J., Newman A.J., Cheng S.C., Abelson J. (1985). Yeast mRNA splicing in vitro. J. Biol. Chem..

[bib42] Liu L., Query C.C., Konarska M.M. (2007). Opposing classes of prp8 alleles modulate the transition between the catalytic steps of pre-mRNA splicing. Nat. Struct. Mol. Biol..

[bib43] Liu Y.C., Chen H.C., Wu N.Y., Cheng S.C. (2007). A novel splicing factor, Yju2, is associated with NTC and acts after Prp2 in promoting the first catalytic reaction of pre-mRNA splicing. Mol. Cell. Biol..

[bib44] Liu S., Li X., Zhang L., Jiang J., Hill R.C., Cui Y., Hansen K.C., Zhou Z.H., Zhao R. (2017). Structure of the yeast spliceosomal postcatalytic P complex. Science.

[bib45] Madhani H.D., Guthrie C. (1994). Genetic interactions between the yeast RNA helicase homolog Prp16 and spliceosomal snRNAs identify candidate ligands for the Prp16 RNA-dependent ATPase. Genetics.

[bib46] Manigrasso J., Chillón I., Genna V., Vidossich P., Somarowthu S., Pyle A.M., De Vivo M., Marcia M. (2020). Visualizing group II intron dynamics between the first and second steps of splicing. Nat. Commun..

[bib47] Marcia M., Pyle A.M. (2012). Visualizing group II intron catalysis through the stages of splicing. Cell.

[bib48] Mayas R.M., Maita H., Staley J.P. (2006). Exon ligation is proofread by the DExD/H-box ATPase Prp22p. Nat. Struct. Mol. Biol..

[bib49] Mayerle M., Guthrie C. (2017). Genetics and biochemistry remain essential in the structural era of the spliceosome. Methods.

[bib50] McPheeters D.S. (1996). Interactions of the yeast U6 RNA with the pre-mRNA branch site. RNA.

[bib51] Newman A.J., Norman C. (1992). U5 snRNA interacts with exon sequences at 5′ and 3′ splice sites. Cell.

[bib52] Nguyen T.H.D., Li J., Galej W.P., Oshikane H., Newman A.J., Nagai K. (2013). Structural basis of Brr2-Prp8 interactions and implications for U5 snRNP biogenesis and the spliceosome active site. Structure.

[bib53] Nguyen T.H.D., Galej W.P., Bai X.-C., Savva C.G., Newman A.J., Scheres S.H.W., Nagai K. (2015). The architecture of the spliceosomal U4/U6.U5 tri-snRNP. Nature.

[bib54] Ohrt T., Odenwälder P., Dannenberg J., Prior M., Warkocki Z., Schmitzová J., Karaduman R., Gregor I., Enderlein J., Fabrizio P., Lührmann R. (2013). Molecular dissection of step 2 catalysis of yeast pre-mRNA splicing investigated in a purified system. RNA.

[bib55] Pineda J.M.B., Bradley R.K. (2018). Most human introns are recognized via multiple and tissue-specific branchpoints. Genes Dev..

[bib56] Plaschka C., Lin P.-C., Nagai K. (2017). Structure of a pre-catalytic spliceosome. Nature.

[bib57] Plaschka C., Newman A.J., Nagai K. (2019). Structural Basis of Nuclear pre-mRNA Splicing: Lessons from Yeast. Cold Spring Harb. Perspect. Biol..

[bib58] Popenda M., Szachniuk M., Antczak M., Purzycka K.J., Lukasiak P., Bartol N., Blazewicz J., Adamiak R.W. (2012). Automated 3D structure composition for large RNAs. Nucleic Acids Res..

[bib59] Price S.R., Evans P.R., Nagai K. (1998). Crystal structure of the spliceosomal U2B″-U2A′ protein complex bound to a fragment of U2 small nuclear RNA. Nature.

[bib60] Query C.C., Konarska M.M. (2004). Suppression of multiple substrate mutations by spliceosomal prp8 alleles suggests functional correlations with ribosomal ambiguity mutants. Mol. Cell.

[bib61] Ramlaul K., Palmer C.M., Nakane T., Aylett C.H.S. (2020). Mitigating local over-fitting during single particle reconstruction with SIDESPLITTER. J. Struct. Biol..

[bib62] Rauhut R., Fabrizio P., Dybkov O., Hartmuth K., Pena V., Chari A., Kumar V., Lee C.-T., Urlaub H., Kastner B. (2016). Molecular architecture of the Saccharomyces cerevisiae activated spliceosome. Science.

[bib63] Robart A.R., Chan R.T., Peters J.K., Rajashankar K.R., Toor N. (2014). Crystal structure of a eukaryotic group II intron lariat. Nature.

[bib64] Scheres S.H.W. (2014). Beam-induced motion correction for sub-megadalton cryo-EM particles. eLife.

[bib65] Schwer B., Guthrie C. (1992). A conformational rearrangement in the spliceosome is dependent on PRP16 and ATP hydrolysis. EMBO J..

[bib66] Semlow D.R., Blanco M.R., Walter N.G., Staley J.P. (2016). Spliceosomal DEAH-Box ATPases Remodel Pre-mRNA to Activate Alternative Splice Sites. Cell.

[bib67] Shannon R.D. (1976). Revised effective ionic radii and systematic studies of interatomic distances in halides and chalcogenides. Acta Crystallogr. A.

[bib68] Sontheimer E.J., Steitz J.A. (1993). The U5 and U6 small nuclear RNAs as active site components of the spliceosome. Science.

[bib69] Steitz T.A., Steitz J.A. (1993). A general two-metal-ion mechanism for catalytic RNA. Proc. Natl. Acad. Sci. USA.

[bib70] Taggart A.J., Lin C.-L., Shrestha B., Heintzelman C., Kim S., Fairbrother W.G. (2017). Large-scale analysis of branchpoint usage across species and cell lines. Genome Res..

[bib71] Terwilliger T.C., Ludtke S.J., Read R.J., Adams P.D., Afonine P.V. (2020). Improvement of cryo-EM maps by density modification. Nat. Methods.

[bib72] Tseng C.K., Cheng S.C. (2008). Both catalytic steps of nuclear pre-mRNA splicing are reversible. Science.

[bib73] Tseng C.-K., Cheng S.-C. (2013). The spliceosome catalyzes debranching in competition with reverse of the first chemical reaction. RNA.

[bib74] Tseng C.K., Liu H.L., Cheng S.C. (2011). DEAH-box ATPase Prp16 has dual roles in remodeling of the spliceosome in catalytic steps. RNA.

[bib75] Tseng C.-K., Chung C.-S., Chen H.-C., Cheng S.-C. (2017). A central role of Cwc25 in spliceosome dynamics during the catalytic phase of pre-mRNA splicing. RNA.

[bib76] van Roon A.-M.M., Yang J.-C., Mathieu D., Bermel W., Nagai K., Neuhaus D. (2015). ^113^Cd NMR experiments reveal an unusual metal cluster in the solution structure of the yeast splicing protein Bud31p. Angew. Chem. Int. Ed. Engl..

[bib95] Vander Kooi Craig (2010). The Prp19 WD40 Domain Contains a Conserved Protein Interaction Region Essential for Its Function. Structure.

[bib77] Vander Kooi C.W., Ohi M.D., Rosenberg J.A., Oldham M.L., Newcomer M.E., Gould K.L., Chazin W.J. (2006). The Prp19 U-box crystal structure suggests a common dimeric architecture for a class of oligomeric E3 ubiquitin ligases. Biochemistry.

[bib78] Vijayraghavan U., Parker R., Tamm J., Iimura Y., Rossi J., Abelson J., Guthrie C. (1986). Mutations in conserved intron sequences affect multiple steps in the yeast splicing pathway, particularly assembly of the spliceosome. EMBO J..

[bib79] Villa T., Guthrie C. (2005). The Isy1p component of the NineTeen complex interacts with the ATPase Prp16p to regulate the fidelity of pre-mRNA splicing. Genes Dev..

[bib80] Wagner T., Merino F., Stabrin M., Moriya T., Antoni C., Apelbaum A., Hagel P., Sitsel O., Raisch T., Prumbaum D. (2019). SPHIRE-crYOLO is a fast and accurate fully automated particle picker for cryo-EM. Commun. Biol..

[bib81] Wan R., Yan C., Bai R., Huang G., Shi Y. (2016). Structure of a yeast catalytic step I spliceosome at 3.4 Å resolution. Science.

[bib82] Wan R., Bai R., Yan C., Lei J., Shi Y. (2019). Structures of the Catalytically Activated Yeast Spliceosome Reveal the Mechanism of Branching. Cell.

[bib83] Warkocki Z., Odenwälder P., Schmitzová J., Platzmann F., Stark H., Urlaub H., Ficner R., Fabrizio P., Lührmann R. (2009). Reconstitution of both steps of Saccharomyces cerevisiae splicing with purified spliceosomal components. Nat. Struct. Mol. Biol..

[bib84] Wilkinson M.E., Fica S.M., Galej W.P., Norman C.M., Newman A.J., Nagai K. (2017). Postcatalytic spliceosome structure reveals mechanism of 3′-splice site selection. Science.

[bib85] Wilkinson M.E., Kumar A., Casañal A. (2019). Methods for merging data sets in electron cryo-microscopy. Acta Crystallogr. D Struct. Biol..

[bib86] Wilkinson M.E., Charenton C., Nagai K. (2020). RNA Splicing by the Spliceosome. Annu. Rev. Biochem..

[bib87] Yan C., Wan R., Bai R., Huang G., Shi Y. (2016). Structure of a yeast activated spliceosome at 3.5 Å resolution. Science.

[bib88] Yan C., Wan R., Bai R., Huang G., Shi Y. (2017). Structure of a yeast step II catalytically activated spliceosome. Science.

[bib89] Yang J., Yan R., Roy A., Xu D., Poisson J., Zhang Y. (2015). The I-TASSER Suite: protein structure and function prediction. Nat. Methods.

[bib90] Zhang K. (2016). Gctf: Real-time CTF determination and correction. J. Struct. Biol..

[bib91] Zhang X., Schwer B. (1997). Functional and physical interaction between the yeast splicing factors Slu7 and Prp18. Nucleic Acids Res..

[bib92] Zhang X., Yan C., Hang J., Finci L.I., Lei J., Shi Y. (2017). An Atomic Structure of the Human Spliceosome. Cell.

[bib93] Zheng S.Q., Palovcak E., Armache J.-P., Verba K.A., Cheng Y., Agard D.A. (2017). MotionCor2: anisotropic correction of beam-induced motion for improved cryo-electron microscopy. Nat. Methods.

[bib94] Zhou Z., Sim J., Griffith J., Reed R. (2002). Purification and electron microscopic visualization of functional human spliceosomes. Proc. Natl. Acad. Sci. USA.

