# Supplementary material for: Structural basis for conformational equilibrium of the catalytic spliceosome
Source: Mol Cell. 2021 Apr 1;81(7):1439–1452.e9. doi: 10.1016/j.molcel.2021.02.021 (PMC8022279; doi:10.1016/j.molcel.2021.02.021)
Supplement: Document S1. Figures S1–S7 and Table S1 [file mmc1.pdf]

Molecular Cell, Volume *81*

Supplemental information

Structural basis for conformational equilibrium of the catalytic spliceosome

Max E. Wilkinson, Sebastian M. Fica, Wojciech P. Galej, and Kiyoshi Nagai

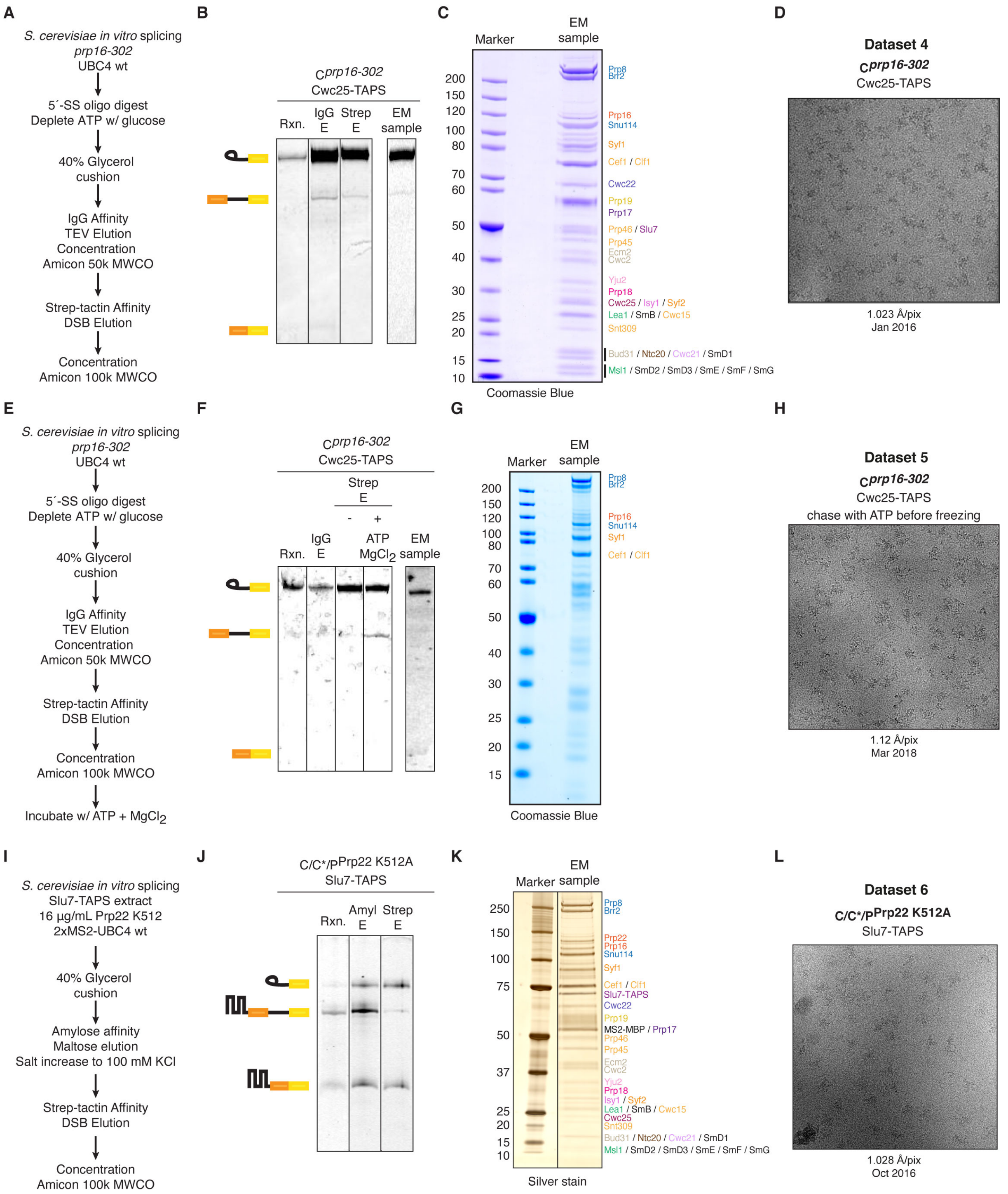

Figure S1, related to Figure 1. Purification and cryo-EM of new spliceosome samples used for this study. (A-D) Purification scheme, RNA and protein composition, and representative micrograph for C-complex Dataset 4. (E-H) Purification scheme, RNA and protein composition, and representative micrograph for C-complex Dataset 5. (I-L) Purification scheme, RNA and protein composition, and representative micrograph for the P-complex Dataset 6. See STAR Methods for details.

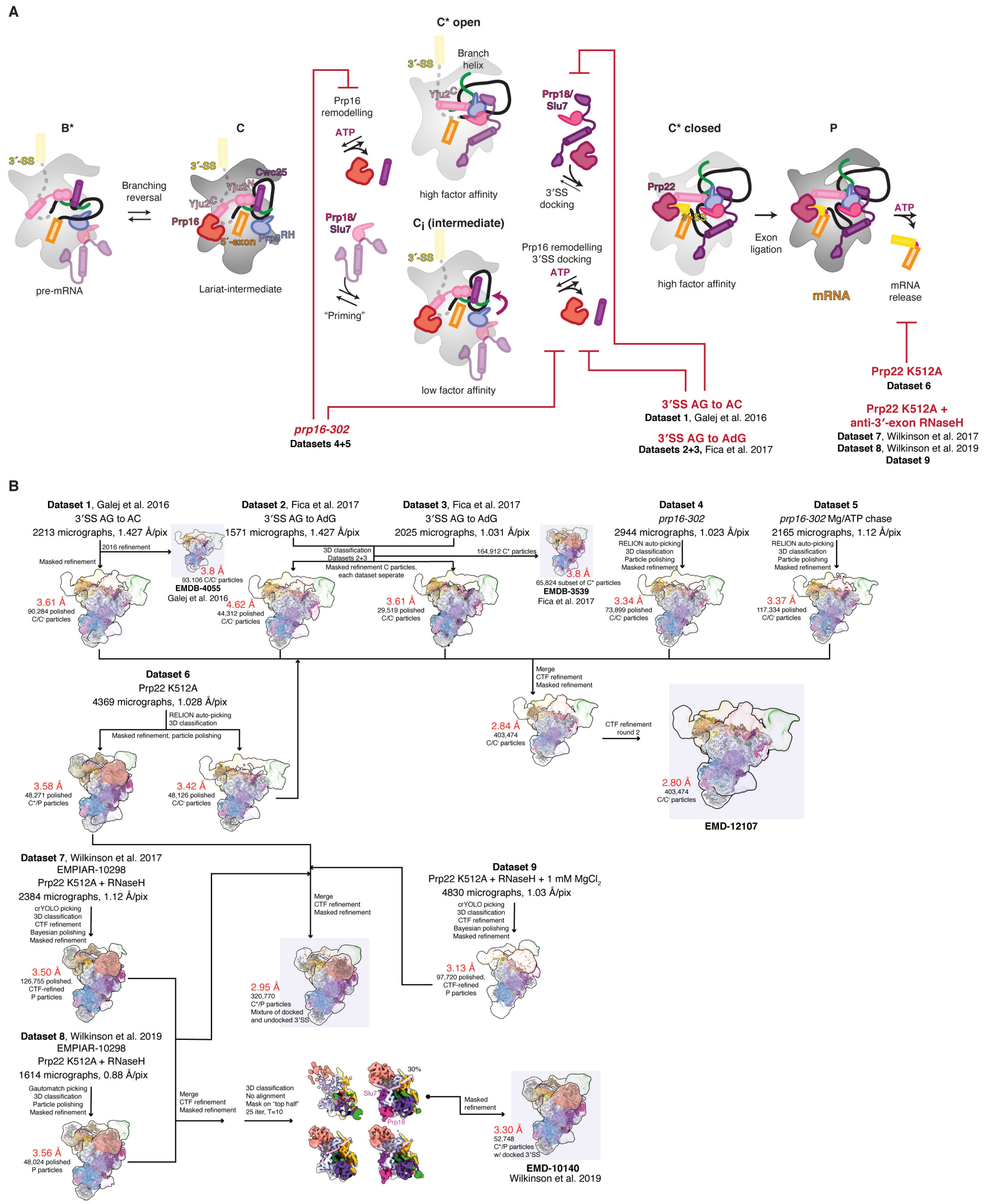

Figure S2, related to Figure 1. Cryo-EM data processing scheme for dataset merging. (A) Biochemical scheme for stalling of spliceosomes used for specific datasets. (B) Dataset merging scheme for producing overall C- and P-complex structures. Final maps deposited to the EMDB are highlighted.

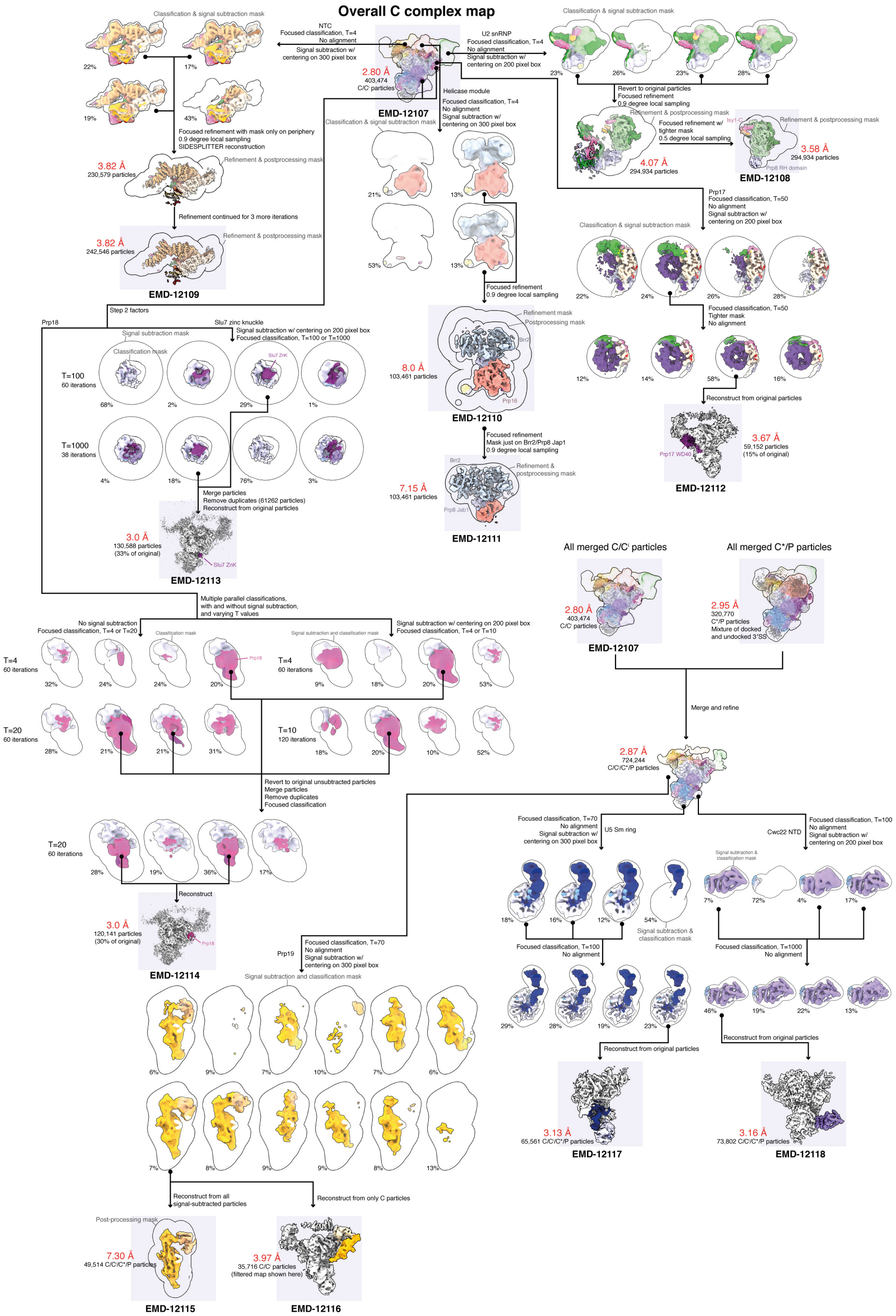

Figure S3, related to Figure 1. Focussed classification scheme for peripheral regions of the C complex. Final maps deposited to the EMDB are highlighted.

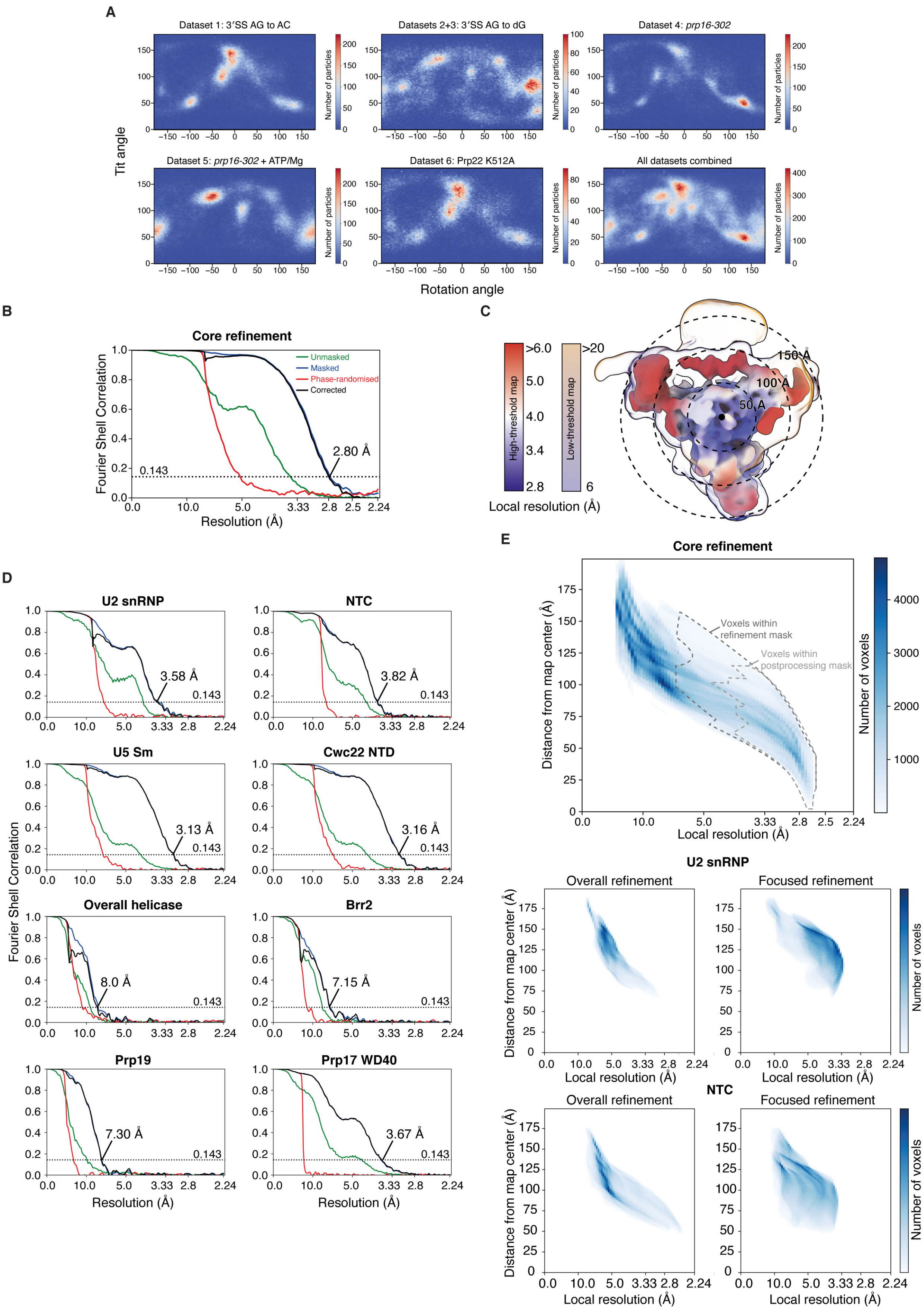

Figure S4, related to Figure 1. Statistics for the cryo-EM datasets and the resulting maps. (A) Orientational distribution for the C-complex cryo-EM datasets. Merging datasets improves the overall angular distribution. (B) Gold-standard Fourier-Shell Correlation curves for the overall C-complex reconstruction and the focus-refined or classified maps. (C) Overall C-complex reconstruction coloured by local resolution. The map is shown at two thresholds coloured with different palettes to show the high core resolution and low peripheral resolution. (D) Heat map of local resolution against distance from map centre, showing how in the overall C-complex reconstruction the local resolution decreases towards the map periphery. (E) Local resolution heat maps for the U2 snRNP and NTC before and after focussed refinement. Focussed refinement improves the resolution distribution.

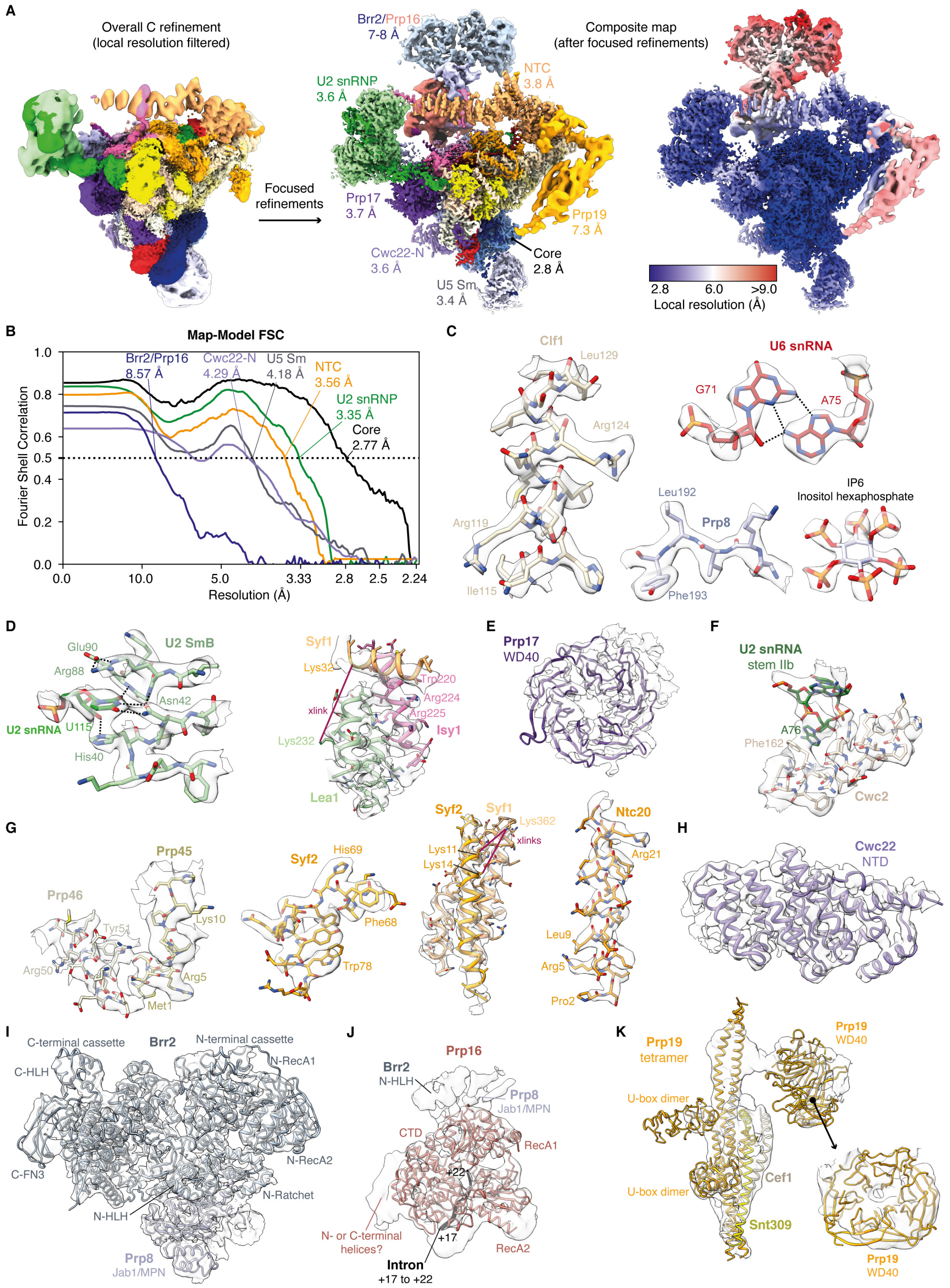

Figure S5, related to Figure 1. Representative cryo-EM densities for the C complex. (A) The overall C-complex reconstruction and a composite map of all focussed refinements. Local resolution for each focus-refined map was first calculated in RELION. Individual local-resolution maps were then resampled in Chimera on the overall reconstruction, and at each voxel the local-resolution map with the lowest value (i.e. best resolution) was taken. (B) FSC curves calculated in PHENIX for each subcomponent of the model (see Table 1) against its respective focus-refined map. (C) Representative densities after density modification for proteins and ligands in the C-complex core. (D-H) Densities for the focus-refined density-modified maps of the U2 snRNP (D), Prp17 WD40 domain, which unambiguously identifies orientation (E), Cwc2 (F), NTC components (G), and for the Cwc22 NTD (H). All crosslinks shown are from the B^{act} complex (Rauhut et al., 2016). (I) Focus-refined Brr2 map. (J) Focus-refined overall helicase module map around Prp16. (K) Focus-classified map of the Prp19 tetramer.

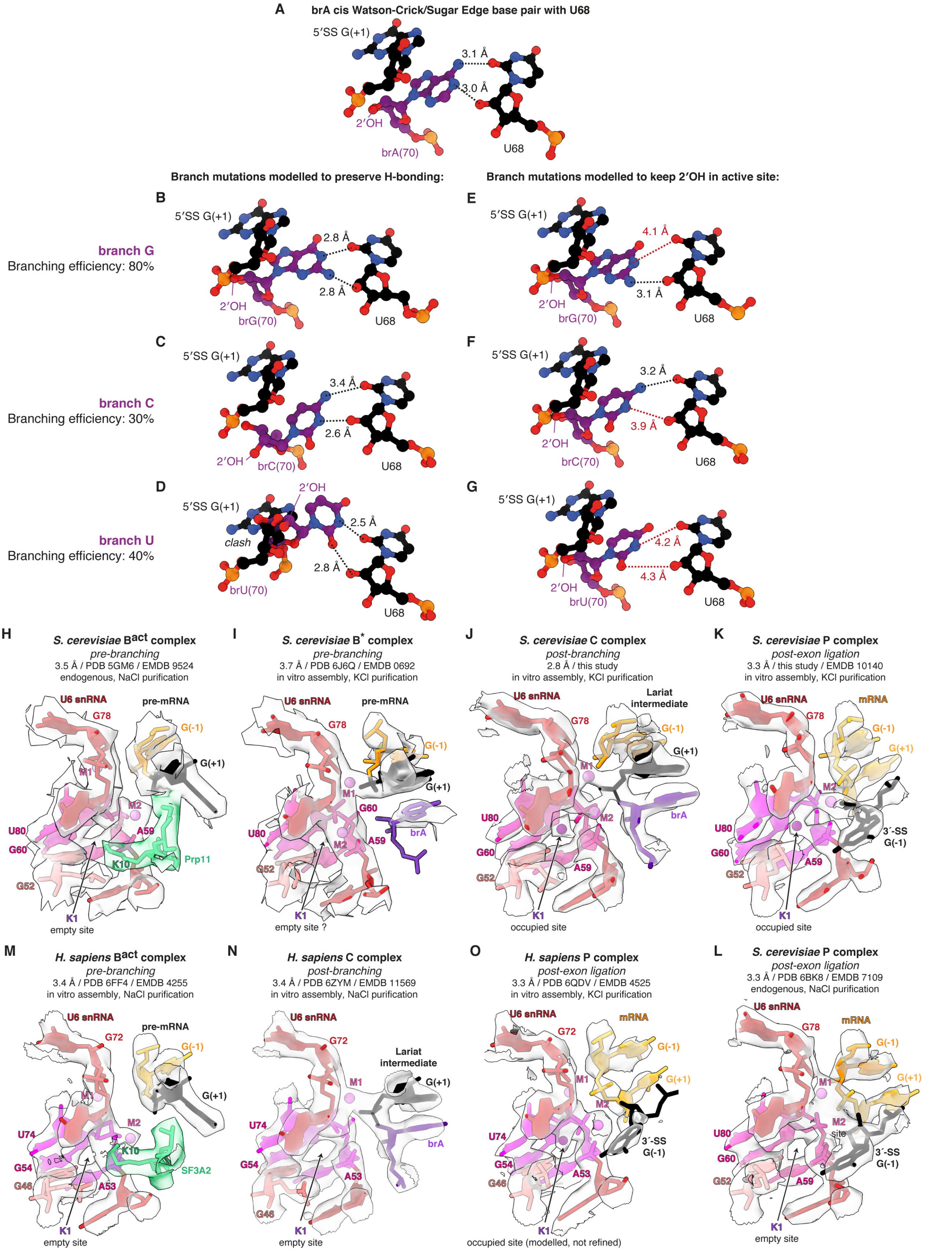

Figure S6, related to Figure 2. Recognition of brA in C complex and comparison of key active site elements of the different spliceosomal complexes. (A) Proposed model for brA interactions in C complex, as derived from this study. (B-G) Modelling of analogous interactions between mutations at brA and U68 of the intron. Models B-D are based on previously documented analogous base pairs (Leontis et al., 2002). Models E-G are simple mutations of the brA base, keeping the sugar and phosphate in the starting position. Branch G can accommodate base pairing and correct 2′-OH positioning, while branch C and U can only accommodate either pairing or correct 2′OH positioning. (H-L) Structure of the M1-M2-K1 catalytic metal cluster in various *S. cerevisiae* spliceosome complexes. The K1 site is partially blocked in B^{act} by Prp11. Potassium is likely absent at the K1 site in the structure of the P complex assembled endogenously due to use of NaCl during purification. (M-O) Structure of the M1-M2-K1 metal cluster in various *H. sapiens* spliceosome complexes. The K1 site is partially blocked in B^{act} by SF3A2, the Prp11 homolog. Clear density for potassium is not resolved at the K1 site in the structure of the human C complex assembled endogenously due to lower resolution and the use of NaCl during purification. For clarity, potassium is represented using 0.5x of its Van der Waals radius, while 1x of its van der Waals radius was used for magnesium.

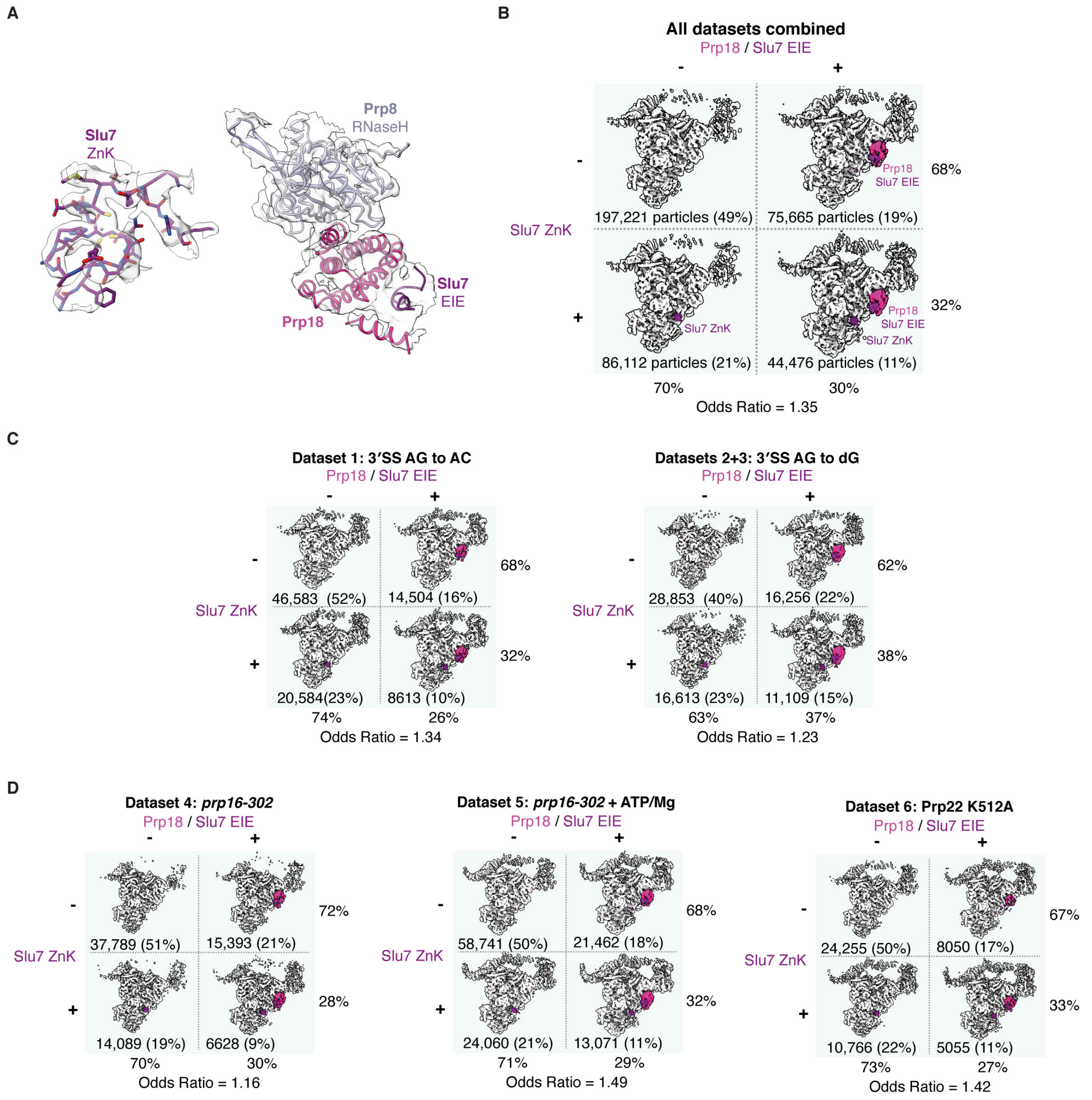

Figure S7, related to Figure 3. Focussed classification of C-complex particles for the C_i conformation. (A) Density quality for Prp18 and Slu7 ZnK and EIE domains in the final map obtained by classification of all datasets. (B) Focused classification of Prp18 and the Slu7 ZnK for all combined C-complex datasets. (C) Focused classification of Prp18 and the Slu7 ZnK for C-complex datasets resulting from impairment of exon ligation by mutation of the 3′-SS. (D) Focused classification of Prp18 and the Slu7 ZnK for C-complex datasets resulting from impairment of exon ligation by mutation of Prp16 or Prp22.

Table S1, Related to Fig. 1. Cryo-EM data processing, refinement, and validation statistics

Map and Model	C-complex Core	U2 snRNP	NTC
**Data processing**			
Number of particle images	403,474	294,934	242,546
Map resolution (FSC=0.143) (Å)[a]	2.80	3.58	3.82
Map resolution range[b] (Å)	2.71 – 4.94	3.39 – 8.20	3.64 – 8.68
Map resolution after density modification (Å)	2.69	3.26	3.50
**Refinement**			
Refined residues[c]	/6/E/I/C/J/K/N/M/P/L/F/a/c2:4-106/5:27-126/A:126-2078,2500/O:11-252/S:36-256/y:95-211/R:2-26/H:289-486/o:51-76/c:122-140,400/G:2-98/D:1-210	/2:107-1109/Y/W/q/p/r/n/k/l/m/G:214-232	/T/S:257-625/y:2-81/Z/G:163-191/K:1-11/J:46-59/X/O:304-327/D:209-225
Model resolution (FSC=0.5) (Å)	2.79	3.34	3.58
Map CC (around atoms)	0.70	0.75	0.69
Map sharpening *B* factor (Å^2)	-89	-138	-157
Model composition			
Non-hydrogen atoms	51,675	9959	8971
Protein residues	5463	932	1260
RNA residues	342	119	0
Ligands	17	0	0
Mean *B* factors (Å^2)			
Protein	100.3	59.0	93.7
RNA	129.7	182.9	
Ligand	104.8		
Root mean square deviations			
Bond lengths (Å)	0.009	0.010	0.011
Bond angles (°)	1.392	1.658	1.457
Validation			
MolProbity score	0.54	0.89	0.79
Clashscore	0.10	0.21	0.30
Rotamer outliers (%)	0.08	0.71	0.14
CaBLAM outliers (%)	0.76	1.25	0.87
RNA geometry			
Correct sugar puckers (%)	95.0	97.5	
Good backbone conformations (%)	80.1	79.8	
Ramachandran plot			
Favoured (%)	98.40	95.58	96.94
Allowed (%)	1.59	4.42	2.97
Disallowed (%)	0.02	0.00	0.09
**Data deposition**			
EMDB (map)	EMD-12107	EMD-12108	EMD-12109
Model (PDB)	7B9V	7B9V	7B9V

[a] FSC, Fourrier Shell Correlation.
[b] Range from 5th to 95th percentiles of local resolution map within the refinement mask
[c] Shown in ChimeraX selection notation, where / indicates chain ID and : indicates residue IDs, e.g. /2:4-47 is chain 2 (U2 snRNA) residues 4 – 47.

Table S1, Related to Fig. 1 (continued). Cryo-EM data collection, refinement and validation statistics

Map and Model	U5 Sm	Cwc22 NTD	Helicase module
**Data processing**			
Number of particle images	65,561	73,802	103,461
Map resolution (FSC=0.143)[a] (Å)	3.13 overall<br>3.4 around U5 Sm	3.16 overall<br>3.6 around Cwc22N	7.15<br>8.0 (composite map)
Map resolution range[b] (Å)	3.06 – 8.41<br>around U5 Sm	3.40 – 5.24<br>around Cwc22N	6.43 – 10.56
Map resolution after density modification (Å)	2.96	2.97	
**Refinement**			
Refined residues[c]	/5:1-26,127-178/b/d/e/f/g/h/j	/H:10-264/X:1-20	/A:2150-2395/B/Q/I:87-92
Model resolution (FSC=0.5)[c] (Å)	4.17	4.25	8.23
Map CC (around atoms)	0.61	0.64	0.60
Map sharpening *B* factor (Å^2)	-74	-89	-275, -494
Model composition			
Non-hydrogen atoms	6424	2166	20,728
Protein residues	602	273	2576
RNA residues	78	0	6
Ligands	0	0	0
Mean *B* factors (Å^2)			
Protein	151.5	154.39	Not refined
RNA	237.3		Not refined
Ligand			
Root mean square deviations			
Bond lengths (Å)	0.01	0.010	0.013
Bond angles (°)	1.600	1.538	1.730
Validation			
MolProbity score	0.92	0.81	0.83
Clashscore	0.41	0.70	0.29
Rotamer outliers (%)	0.19	0.00	0.17
CaBLAM outliers (%)	0.88	0.40	1.49
RNA geometry			
Correct sugar puckers (%)	93.6		83.3
Good backbone conformations (%)	71.8		83.3
Ramachandran plot			
Favoured (%)	96.06	97.62	96.61
Allowed (%)	3.77	2.38	3.39
Disallowed (%)	0.17	0.00	0.00
**Data deposition**			
EMDB (map)	EMD-12117	EMD-12118	EMD-12110<br>EMD-12111
Model (PDB)	7B9V	7B9V	7B9V

[a] FSC, Fourrier Shell Correlation.
[b] Range from 5th to 95th percentiles of local resolution map within the refinement mask
[c] Shown in ChimeraX selection notation, where / indicates chain ID and : indicates residue IDs, e.g. /2:4-47 is chain 2 (U2 snRNA) residues 4 – 47.

Table S1, Related to Fig. 1 (continued). Cryo-EM data collection, refinement and validation statistics

**Map and Model**	Prp17 WD40	Prp19	C_i-complex Composite
**Data processing**			
Particle images (no.)	59,152	49,514	NA
Map resolution (FSC=0.143)[a] (Å)	3.67 overall	7.30	Composite map
Map resolution range[b] (Å)	3.27 – 4.74 around Prp17	6.79 – 9.48	2.85 – 10.6
**Refinement**			
Refined residues[c]	/o:149-454	/O:484-536/s/t/u/v/w/X:35-51	All
Model resolution (FSC=0.5) (Å)	N.D.	N.D.	3.1
Map CC (around atoms)	0.43	0.39	N.D.
Map sharpening *B* factor (Å^2)	-90	-200	-
Model composition			
Non-hydrogen atoms	2454	6729	109,106
Protein residues	304	906	12,316
RNA residues	0	0	545
Ligands	0	0	17
Mean *B* factors (Å^2)			
Protein	Not refined	Not refined	134.09
RNA			156.50
Ligand			104.41
Root mean square deviations			
Bond lengths (Å)	0.011	0.012	0.010
Bond angles (°)	1.883	1.827	1.547
Validation			
MolProbity score	0.93	0.78	0.71
Clashscore	0.21	0.91	0.25
Rotamer outliers (%)	0.36	0.15	0.17
CaBLAM outliers (%)	2.70	0.36	0.98
RNA geometry			
Correct sugar puckers (%)			95.0
Good backbone conformations (%)			78.5
Ramachandran plot			
Favoured (%)	95.00	98.25	97.44
Allowed (%)	4.67	1.51	2.51
Disallowed (%)	0.33	0.23	0.05
**Data deposition**			
EMDB (map)	EMD-12112	EMD-12115	EMD-12106
Model (PDB)	7B9V	7B9V	7B9V

[a] FSC, Fourrier Shell Correlation.

[b] Range from 5th to 95th percentiles of local resolution map within the refinement mask

[c] Shown in ChimeraX selection notation, where / indicates chain ID and : indicates residue IDs, e.g. /2:4-47 is chain 2 (U2 snRNA) residues 4 – 47.
